# Development of New Antimicrobial Oleanonic Acid Polyamine Conjugates

**DOI:** 10.3390/antibiotics11010094

**Published:** 2022-01-12

**Authors:** Elmira F. Khusnutdinova, Véronique Sinou, Denis A. Babkov, Oxana Kazakova, Jean Michel Brunel

**Affiliations:** 1 Ufa Institute of Chemistry UFRC RAS, 71 pr. Oktyabrya, 450054 Ufa, Russia; obf@anrb.ru; 2 Aix Marseille Univ, INSERM, SSA, MCT, 13385 Marseille, France; veronique.sinou@univ-amu.fr; 3 Scientific Center for Innovative Drugs, Volgograd State Medical University, Novorossiyskaya st. 39, 400087 Volgograd, Russia; denis.a.babkov@gmail.com

**Keywords:** oleanolic acid, triterpenic polyamine conjugates, antimicrobial activities, antibiotic enhancers

## Abstract

A series of oleanolic acid derivatives holding oxo- or 3-*N*-polyamino-3-deoxy-substituents at C3 as well as carboxamide function at C17 with different long chain polyamines have been synthesized and evaluated for antimicrobial activities. Almost all series presented good to moderate activity against Gram-positive *S. aureus*, *S. faecalis* and *B. cereus* bacteria with minimum inhibitory concentration (MIC) values from 3.125 to 200 µg/mL. Moreover, compounds possess important antimicrobial activities against Gram-negative *E. coli*, *P. aeruginosa*, *S. enterica*, and *EA289* bacteria with MICs ranging from 6.25 to 200 µg/mL. The testing of ability to restore antibiotic activity of doxycycline and erythromycin at a 2 µg/mL concentration in a synergistic assay showed that oleanonic acid conjugate with spermine spacered through propargylamide led to a moderate improvement in terms of antimicrobial activities of the different selected combinations against both *P. aeruginosa* and *E. coli*. The study of mechanism of action of the lead conjugate **2i** presenting a *N*-methyl norspermidine moiety showed the effect of disruption of the outer bacterial membrane of *P. aeruginosa* PA01 cells. Computational ADMET profiling renders this compound as a suitable starting point for pharmacokinetic optimization. These results give confidence to the successful outcome of bioconjugation of polyamines and oleanane-type triterpenoids in the development of antimicrobial agents.

## 1. Introduction

Multidrug resistance (MDR) to antibiotics leads to serious issues in the treatment of microbial infections, which means an extremely high need for the search and development of new antimicrobial agents [1]. One solution consists of combining therapies of existing antibiotics with potentiating adjuvants (chemosensitizer agents), which re-empower the antibiotic agents to become efficacious against the resistant strains [2]. These compounds do not directly kill bacteria but enhance antibiotic activity by inhibiting antibiotic-modifying enzymes or by increasing the intracellular antibiotic accumulation through inhibiting efflux pumps, as well as facilitating the permeation of antibiotics entrance across membranes. Adjuvants can also target the biofilm formation by inhibiting the signaling and regulatory pathways that mediate antibiotic resistance as well as enhance the host defense by stimulating the immune cells [3]. The pharmaceutical market contains primarily antibiotics obtained from natural substances by means of semi-synthetic modifications and synthetic analogs of natural antibiotics [4]. 

Today, development of natural products for the prevention and treatment of diseases continue to attract attention worldwide [5,6]. Among them, plant-derived triterpenes represent an interesting class of molecules with a multitude of activities that make them references for drug-discovery programs as proven by the numerous ongoing clinical trials and drugs on the market. For example, the lupane-type triterpene betulin, which is the main component of birch bark extract, has medicinal properties for epidermolysis bullosa, wounds and burns [7]. Betulinic acid, which comes from oxidation of betulin, is under clinical trials for the treatment of dysplastic nevus syndrome and psychological stress [8], and boswellic acid, isolated from *Boswellia serrata*, is under trial for treatment of relapsing remitting multiple sclerosis, renal stones, joint pain and stiffness [9]. The synthetic derivative of oleanolic acid CDDO-Me demonstrated its efficacy as anticancer drug in different mouse models, and versus several types of cancer [10]. Ursolic acid, isolated from plants as *Rosmarinus officinalis*, *Malus domestica*, *Salvia officinalis*, and *Thymus vulgarisis,* is under clinical trials for use against metabolic syndrome and sarcopenia [11].

In recent years, triterpenes were identified as a promising class for the development of new antibiotics, as they are active against antibiotic-resistant strains [12]. The most complete picture of the antimicrobial properties of triterpenoids is presented for the *Staphylococcus aureus* culture, native and semi-synthetic derivatives of the ursane, oleanane, and lupane series being identified as the most promising [13]. Although there is no specific report on their modes of action, the inhibition of *S. aureus* growth through novel targets should stimulate research to develop triterpenic acids as effective agents against *S. aureus* bacteria [14]. Furthermore, it was shown that ursolic acid has a synergistic effect when associated with ampicillin and tetracycline against both *Bacillus cereus* and *S. aureus* [15]. Moreover, oleanolic and ursolic acids may be useful when administered in combination with β-lactam antibiotics to combat bacterial infections caused by some Gram-positive pathogens [16].

There are limited data about the antimicrobial activity of semi-synthetic derivatives derived from native triterpenoids [17]. On the other hand, polyamines conjugates are becoming important in all the biological and medicinal fields [18]. It is shown [19] that C3 or C28 functionalization of betulinic acid with the formation of polyamine or polyarginine derivatives (or their shorter analogs [20]) can significantly increase the MIC index ranging from 3.125–12.5 and 3.9–7.8 mg/L for *S. aureus*, respectively. Spermine derivatives of heterobetulonic and ursolic acids displayed antimicrobial activity on *S. aureus*, *Streptococcus mutans* and *Listeria monocytogenes* at a concentration of 6.25 mM, and cytotoxicity on different cancer cell lines and were characterized as supramolecular systems [21]. The amphiphile-like oleanolic acid-triazole-spermine conjugates self-assemble into highly entangled fibrous networks leading to gelation which can be used for multifunctional soft organic nanomaterials [22]. Triterpenic polyamines being analogs of steroidal squalamine and trodusquemine, were found as important substances for the search of new drugs with anticancer, antidiabetic and antimicrobial activities [23]. Oleanolic acid conjugates with homopyperazine, and diethylenetriamine fragments demonstrated a high inhibitory activity against *C. trachomatis* with a chemotherapeutic index (CTI) of 8 and >8, respectively [24].

In this context, we report herein the synthesis, biological evaluation, and structure-activity relationship analysis of a series of oleanane diamino- and polyamino-derivatives, the evaluation of their antimicrobial activities against multidrug-resistant Gram-negative and Gram-positive bacteria, including antibiotic enhancer potency, as well as the study of mechanism of action of the best compound.

## 2. Experimental Section

All the solvents and reagents used were commercially available. Methanol, ethyl acetate, and dichloromethane were purchased from Sigma-Aldrich and used without further purification. Column chromatography was performed on Merck silica gel (70–230 mesh). ^1^H NMR and ^13^C NMR spectra were recorded in MeOD on a Bruker AC 300 spectrometer working at 300 and 75 MHz, respectively (the usual abbreviations are used: s: singlet, d: doublet, t: triplet, q: quadruplet, m: multiplet). All chemical shifts are given in ppm. Mass spectroscopy analysis has been performed by the LC-MS Agilent with a single quadrupole mass. Compounds were prepared as previously reported: **2a** in [25], **2g** in [26], **5** in [27].

### 2.1. Synthesis of Amide Derivatives ***2a**–**2n***, and ***7a**–**7c***

To a solution of compound **1** (for synthesis of **2a**–**2n**) or oleanolic acid (for synthesis of **7a**–**7c**) (100 mg, 0.2 mmol) in 20 mL of DCM, the corresponding amine (0.2 mmol), BOP reagent (88 mg, 0.2 mmol) and DIPEA (0.14 mL, 0.8 mmol) were added, and the resulting mixture was stirred at room temperature for 12 h. After the reaction was completed (as indicated also by TLC), a solution of saturated ammonium chloride (1 mL) was added and the mixture was stirred at room temperature for 2 h. After the organic layer was washed with same solution of ammonium chloride (1 mL × 2), water layer was extracted with ethyl acetate (5 mL), and the combined organic layer was washed using saturated solution of sodium chloride (10 mL × 2) and evaporated under reduced pressure. The crude amide was purified by flash chromatography on silicagel using CH_2_Cl_2_/MeOH/NH_4_OH (7/3/1) as the eluent affording the expected coupling product **2a**–**2n**, and **7a**–**7c** (see Supplementary Materials).

*N*-(3-aminopropyl)-3-oxo-olean-12-en-28-amide (**2b**). 68%, yield; ^1^H NMR (MeOH/CDCl_3_, MHz): 0.49 (s, 3H, H-30), 0.58 (s, 3H, H-23), 0.60 (s, 3H, H-26), 0.72 (s, 3H, H-29), 0.74 (s, 3H, H-24), 0.76 (s, 3H, H-27), 0.85 (s, 3H, H-25), 0.86–2.30 (m, 25H, CH and CH_2_), 2.30–2.48 (m, 2H, CH_2_NH_2_), 2.81–3.15 (m, 2H, NHCH_2_), 2.88–3.05 (m, 3H, NH and NH_2_), 5.08 (s, 1H, H-12). ^13^C NMR (MeOH, MHz): 14.3, 16.3, 20.7, 21.5, 22.7, 23.4, 24.4, 25.1, 30.1, 30.5, 31.5, 32.0, 32.3, 33.6, 36.0, 36.3, 37.3, 37.8, 38.9, 39.0, 41.1, 41.5, 45.9, 47.0, 47.3, 47.5, 47.7, 48.2, 54.8, 122.2 (C12), 143.7 (C13), 179.2 (C28), 216.2. ESI-MS: *m*/*z* = 511 [M + H^+^].

*N*-(5-aminopentyl)-3-oxo-olean-12-en-28-amide (**2c**). 70%, yield; ^1^H NMR (MeOH, MHz): 0.84 (s, 3H, H-30), 0.93 (s, 3H, H-23), 0.97 (s, 3H, H-26), 1.05 (s, 3H, H-29), 1.07 (s, 3H, H-24), 1.09 (s, 3H, H-27), 1.21 (s, 3H, H-25), 1.31–2.13 (m, 29H, CH and CH_2_), 2.62–2.68 (m, 2H, CH_2_NH_2_), 2.81–2.91 (m, 2H, NHCH_2_), 3.08–3.28 (m, 3H, NH and NH_2_), 5.38 (s, 1H, H-12). ^13^C NMR (MeOH, MHz): 15.6, 17.8, 20.7, 21.9, 23.9, 24.6, 25.0, 26.3, 26.9, 27.0, 28.5, 29.1, 30.1, 31.6, 33.4, 33.6, 34.4, 35.1, 35.1, 37.0, 37.9, 40.1, 40.7, 42.6, 43.1, 47.5, 47.6, 48.1, 48.5, 56.5, 56.5, 123.7 (C12), 145.4 (C13), 180.3 (C28), 217.5. ESI-MS: *m*/*z* = 539 [M + H^+^]. 

*N*-(3-(2-oxopyrrolidin-1-yl)propyl)-3-oxo-olean-12-en-28-amide (**2d**). 78%, yield; ^1^H NMR (MeOH, MHz): 0.86 (s, 3H, H-30), 1.01 (s, 3H, H-23), 1.05 (s, 3H, H-26), 1.10 (s, 3H, H-29), 1.12 (s, 3H, H-24), 1.14 (s, 3H, H-27), 1.25 (s, 3H, H-25), 1.26–3.26 (m, 32H, CH and CH_2_), 3.38–3.62 (m, 4H, NHCH_2_, CH_2_N), 5.35 (s, 1H, H-12). ^13^C NMR (MeOH, MHz): 15.5, 17.7, 19.7, 21.7, 22.0, 24.1, 26.5, 27.2, 27.6, 28.8, 31.9, 33.7, 35.0, 36.9, 37.3, 37.8, 37.9, 39.7, 40.4, 40.9, 42.5, 42.8, 42.9, 45.0, 46.1, 47.4, 47.5, 48.0, 48.4, 49.9, 56.4, 86.8, 126.6 (C12), 145.0 (C13), 177.9, 179.8 (C28), 217.5. ESI-MS: *m*/*z* = 579 [M + H^+^].

*N*-(3-morpholinopropyl)-3-oxo-olean-12-en-28-amide (**2e**). 80%, yield; ^1^H NMR (MeOH, MHz): 0.85 (s, 3H, H-30), 0.94 (s, 3H, H-23), 0.98 (s, 3H, H-26), 1.09 (s, 3H, H-29), 1.11 (s, 3H, H-24), 1.13 (s, 3H, H-27), 1.23 (s, 3H, H-25), 1.25–2.21 (m, 24H, CH and CH_2_), 2.37–2.46 (m, 4H, (CH_2_)_2_N), 2.49–3.37 (m, 6H, 3CH_2_), 3.63–3.81 (m, 4H, (CH_2_)_2_O), 5.35 (s, 1H, H-12). ^13^C NMR (MeOH, MHz): 15.1, 17.5, 20.3, 21.5, 23.6, 23.6, 24.2, 26.0, 26.5, 26.5, 28.1, 31.2, 32.9, 33.1, 34.7, 36.5, 36.6, 37.5, 38.6, 39.8, 40.2, 42.6, 47.1, 47.2, 47.7, 48.1, 49.4, 54.3, 54.3, 56.1, 57.3, 67.2, 67.2, 123.3 (C12), 145.0 (C13), 179.8 (C28), 217.5. ESI-MS: *m*/*z* = 581 [M + H^+^].

*N*-(3-(4-(3-aminopropyl)piperazin-1-yl)propyl)-3-oxo-olean-12-en-28-amide (**2f**). 75%, yield; ^1^H NMR (MeOH, MHz): 0.86 (s, 3H, H-30), 0.93 (s, 3H, H-23), 0.98 (s, 3H, H-26), 1.09 (s, 3H, H-29), 1.10 (s, 3H, H-24), 1.12 (s, 3H, H-27), 1.24 (s, 3H, H-25), 1.24–2.15 (m, 31H, CH and CH_2_), 2.16–2.62 (m, 8H, 4CH_2_), 2.38–2.51 (m, 2H, CH_2_NH_2_), 2.78–2.88 (m, 2H, NHCH_2_), 3.09–3.29 (m, 3H, NH and NH_2_), 5.38 (s, 1H, H-12). ^13^C NMR (MeOH, MHz): 15.5, 17.9, 20.7, 21.9, 23.8, 24.0, 24.1, 24.6, 25.1, 26.4, 27.0, 27.1, 27.9, 28.6, 30.7, 31.6, 33.3, 33.5, 34.4, 35.1, 37.9, 39.0, 40.1, 40.2, 40.7, 42.6, 43.1, 47.5, 48.1, 48.5, 48.6, 53.6, 53.9, 56.5, 56.8, 57.2, 123.8 (C12), 145.4 (C13), 180.2 (C28), 217.5. ESI-MS: *m*/*z* = 637 [M + H^+^].

*N*-(3-((3-aminopropyl)amino)propyl)-3-oxo-olean-12-en-28-amide (**2h**). 62%, yield; ^1^H NMR (MeOH, MHz): 0.87 (s, 3H, H-30), 0.92 (s, 3H, H-23), 0.98 (s, 3H, H-26), 1.05 (s, 3H, H-29), 1.09 (s, 3H, H-24), 1.11 (s, 3H, H-27), 1.23 (s, 3H, H-25), 1.24–2.12 (m, 25H, CH and CH_2_), 2.31–3.40 (m, 10H, 5CH_2_), 3.12–3.32 (m, 4H, 2NH and NH_2_), 5.39 (s, 1H, H-12). ^13^C NMR (MeOH, MHz): 15.5, 15.6, 17.9, 20.7, 21.9, 22.0, 24.0, 24.1, 24.6, 26.4, 27.0, 27.4, 28.6, 30.0, 31.7, 33.6, 34.5, 35.1, 37.0, 37.0, 37.9, 38.6, 40.2, 40.7, 42.6, 43.1, 45.5, 47.6, 48.1, 48.5, 53.8, 56.5, 123.8 (C12), 145.4 (C13), 180.3 (C28), 217.5. ESI-MS: *m*/*z* = 568 [M + H^+^].

*N*-(3-((3-aminopropyl)(methyl)amino)propyl)-3-oxo-olean-12-en-28-amide (**2i**). 72%, yield; ^1^H NMR (MeOH, MHz): 0.85 (s, 3H, H-30), 0.92 (s, 3H, H-23), 0.97 (s, 3H, H-26), 1.08 (s, 3H, H-29), 1.09 (s, 3H, H-24), 1.11 (s, 3H, H-27), 1.23 (s, 3H, H-25), 1.24–2.15 (m, 23H, CH and CH_2_), 2.25 (s, 3H, NCH_3_), 2.36–2.68 (m, 8H, 4CH_2_), 2.38–2.51 (m, 2H, CH_2_NH_2_), 2.78–2.88 (m, 2H, NHCH_2_), 3.09–3.29 (m, 3H, NH and NH_2_), 5.38 (s, 1H, H-12). ^13^C NMR (MeOH, MHz): 14.2, 16.5, 19.3, 20.6, 22.6, 22.7, 23.3, 25.1, 25.7, 26.1, 27.3, 30.2, 31.9, 32.2, 33.0, 33.7, 33.8, 36.5, 37.7, 37.8, 38.8, 39.3, 40.9, 41.2, 41.7, 46.2, 46.7, 47.1, 47.2, 47.3, 47.5, 54.9, 55.1, 122.4 (C12), 144.1 (C13), 179.1 (C28), 217.5. HRMS (ESI-TOF): *m*/*z* ([M + H]^+^) 582.4954.

*N*-(2-((2-((2-((2-aminoethyl)amino)ethyl)amino)ethyl)amino)ethyl)-3-oxo-olean-12-en-28-amide (**2j**). 59%, yield; ^1^H NMR (MeOH, MHz): 0.86 (s, 3H, H-30), 0.93 (s, 3H, H-23), 0.98 (s, 3H, H-26), 1.09 (s, 3H, H-29), 1.10 (s, 3H, H-24), 1.12 (s, 3H, H-27), 1.24 (s, 3H, H-25)), 1.29–2.29 (m, 29H, CH and CH_2_), 2.51–3.15 (m, 16H, CH_2_NH_2_ and NHCH_2_), 5.41 (s, 1H, H-12). ^13^C NMR (MeOH, MHz): 15.7, 17.9, 20.8, 21.9, 22.0, 24.1, 24.7, 26.5, 27.0, 27.9, 28.6, 31.7, 33.3, 33.6, 34.2, 34.4, 35.1, 36.6, 37.4, 37.9, 38.9, 39.5, 40.1, 40.7, 42.6, 43.1, 46.8, 47.6, 47.7, 48.2, 48.5, 54.0, 56.5, 57.7, 123.9, 145.4, 180.5, 217.5. ESI-MS: *m*/*z* = 626 [M + H^+^].

*N-*(14-amino-3,6,9,12-tetraazatetradecyl)-3-oxo-olean-12-en-28-amide (**2k**). 52%, yield; ^1^H NMR (MeOH, MHz): 0.87 (s, 3H, H-30), 0.93 (s, 3H, H-23), 0.97 (s, 3H, H-26), 1.08 (s, 3H, H-29), 1.09 (s, 3H, H-24), 1.11 (s, 3H, H-27), 1.23 (s, 3H, H-25)), 1.26–2.29 (m, 43H, CH and CH_2_), 3.21–3.42 (m, 7H, 5NH, NH_2_), 5.31 (s, 1H, H-12). ^13^C NMR (MeOH, MHz): 15.4, 16.4, 17.6, 18.3, 19.4, 20.5, 21.7, 21.8, 22.4, 23.6, 23.8, 23.9, 24.4, 26.1, 26.2, 26.8, 28.4, 31.4, 32.5, 33.1, 33.4, 34.2, 34.9, 35.5, 36.8, 37.7, 40.0, 40.4, 41.7, 42.4, 42.8, 46.5, 47.4, 47.9, 48.3, 56.3, 123.6 (C12), 145.4 (C13), 179.2 (C28), 217.5. ESI-MS: *m*/*z* = 670 [M + H^+^].

*N*-(3-((4-((3-aminopropyl)amino)butyl)amino)propyl)-3-oxo-olean-12-en-28-amide (**2l**). 60%, yield; ^1^H NMR (MeOH, MHz): 0.86 (s, 3H, H-30), 0.93 (s, 3H, H-23), 0.98 (s, 3H, H-26), 1.09 (s, 3H, H-29), 1.10 (s, 3H, H-24), 1.12 (s, 3H, H-27), 1.23 (s, 3H, H-25), 1.22–2.14 (m, 36H, CH and CH_2_), 2.40–3.31 (m, 12H, 6CH_2_), 5.38 (s, 1H, H-12). ^13^C NMR (MeOH, MHz): 15.6, 16.4, 17.9, 19.9, 20.7, 21.7, 22.0, 24.0, 24.1, 24.6, 26.5, 27.0, 27.0, 28.1, 28.6, 29.9, 31.7, 33.3, 33.6, 34.5, 35.1, 35.1, 37.9, 38.0, 38.5, 40.2, 40.6, 42.6, 42.9, 43.0, 45.1, 46.2, 47.0, 47.5, 47.6, 47.9, 48.1, 48.5, 49.9, 50.4, 56.5, 123.7 (C12), 145.3 (C13), 180.2 (C28), 217.5. ESI-MS: *m*/*z* = 640 [M + H^+^].

*N*-(3-(4-(3-aminopropoxy)butoxy)propyl)-3-oxo-olean-12-en-28-amide (**2m**). 59%, yield; ^1^H NMR (MeOH, MHz): 0.87 (s, 3H, H-30), 0.94 (s, 3H, H-23), 0.99 (s, 3H, H-26), 1.01 (s, 3H, H-29), 1.11 (s, 3H, H-24), 1.12 (s, 3H, H-27), 1.24 (s, 3H, H-25), 1.26–2.80 (m, 34H, CH and CH_2_), 2.98–3.61 (m, 12H, ((CH_2_)_2_O)_2_, NHCH_2_, CH_2_NH_2_), 5.35 (s, 1H, H-12). ^13^C NMR (MeOH, MHz): 15.6, 17.8, 20.7, 21.9, 24.1, 24.6, 26.4, 27.0, 27.5, 27.5, 28.5, 29.2, 30.5, 31.6, 33.3, 33.5, 34.4, 35.1, 37.9, 38.6, 39.3, 40.2, 40.7, 42.7, 43.1, 47.4, 47.5, 47.6, 48.1, 48.5, 48.6, 56.5, 69.4, 70.2, 71.9, 72.0, 123.8 (C12), 145.4 (C13), 180.1 (C28), 218.5. ESI-MS: *m*/*z* = 641 [M + H^+^].

*N*-(3-(bis(3-aminopropyl)amino)propyl)-3-oxo-olean-12-en-28-amide (**2n**). 49%, yield; ^1^H NMR (MeOH, MHz): 0.87 (s, 3H, H-30), 0.94 (s, 3H, H-23), 0.99 (s, 3H, H-26), 1.01 (s, 3H, H-29), 1.11 (s, 3H, H-24), 1.12 (s, 3H, H-27), 1.24 (s, 3H, H-25), 1.25–2.72 (m, 40H, CH and CH_2_), 3.12–3.30 (m, 6H, NHCH_2_, 2CH_2_NH_2_), 5.33 (s, 1H, H-12). ^13^C NMR (MeOH, MHz): 15.6, 15.7, 18.0, 20.7, 21.9, 22.0, 24.0, 24.1, 24.7, 26.4, 26.9, 27.5, 28.6, 30.3, 31.7, 33.4, 33.6, 34.4, 35.2, 37.9, 39.3, 40.1, 40.7, 41.1, 42.6, 43.1, 47.5, 47.6, 48.2, 48.5, 49.0, 49.9, 49.9, 52.7, 56.5, 123.7 (C12), 145.4 (C13), 180.1 (C28), 217.5. ESI-MS: *m*/*z* = 625 [M + H^+^].

*N*-(3-((3-aminopropyl)amino)propyl)-3b-hydroxy-olean-12-en-28-amide (**7a**). 53%, yield; ^1^H NMR (MeOH, MHz): 0.74 (s, 3H, H-30), 0.75 (s, 3H, H-23), 0.82 (s, 3H, H-26), 0.88 (s, 3H, H-29), 0.93 (s, 3H, H-24), 0.95 (s, 3H, H-27), 1.23 (s, 3H, H-25), 1.24–2.15 (m, 26H, CH and CH_2_), 2.31–3.40 (m, 10H, 5CH_2_), 3.12–3.23 (m, 4H, 2NH and NH_2_), 3.25–3.28 (m, 1H, H-3), 5.28 (s, 1H, H-12). ^13^C NMR (MeOH, MHz): 16.2, 16.6, 18.2, 19.7, 24.1, 24.3, 24.8, 26.8, 28.1, 28.8, 29.0, 29.3, 31.8, 33.8, 34.1, 34.8, 35.3, 38.2, 38.4, 39.7, 40.0, 40.0, 40.1, 40.9, 42.7, 43.1, 47.5, 47.6, 47.7, 47.8, 50.1, 56.9, 79.8 (C3), 124.2 (C12), 145.5 (C13), 180.9 (C28). ESI-MS: *m*/*z* = 570 [M + H^+^].

*N*-(3-((3-aminopropyl)(methyl)amino)propyl)-3b-hydroxy-olean-12-en-28-amide (**7b**). 60%, yield; ^1^H NMR (MeOH, MHz): 0.78 (s, 3H, H-30), 0.91 (s, 3H, H-23), 0.92 (s, 3H, H-26), 0.94 (s, 3H, H-29), 1.04 (s, 3H, H-24), 1.06 (s, 3H, H-27), 1.19 (s, 3H, H-25), 1.20–2.21 (m, 24H, CH and CH_2_), 2.84 (s, 3H, NCH_3_), 2.36–2.68 (m, 8H, 4CH_2_), 2.38–2.51 (m, 2H, CH_2_NH_2_), 2.78–2.88 (m, 2H, NHCH_2_), 3.09–3.29 (m, 3H, NH and NH_2_), 3.34–3.36 (m, 1H, H-3), 5.38 (s, 1H, H-12). ^13^C NMR (MeOH, MHz): 15.9, 16.3, 17.9, 19.4, 23.5, 23.9, 24.1, 24.5, 25.7, 26.5, 27.8, 28.5, 28.8, 31.6, 33.3, 33.6, 34.5, 35.1, 37.0, 37.4, 37.9, 38.0, 38.1, 39.8, 40.4, 40.6, 42.3, 42.8, 47.5, 47.6, 48.1, 48.5, 54.3, 79.6 (C3), 124.1 (C12), 144.9 (C13), 181.2 (C28). ESI-MS: *m*/*z* = 584 [M + H^+^].

*N*-(2-((2-((2-((2-aminoethyl)amino)ethyl)amino)ethyl)amino)ethyl)-3b-hydroxy-olean-12-en-28-amide (**7c**). 47%, yield; ^1^H NMR (MeOH, MHz): 0.78 (s, 3H, H-30), 0.80 (s, 3H, H-23), 0.92 (s, 3H, H-26), 0.94 (s, 3H, H-29), 0.98 (s, 3H, H-24), 1.18 (s, 3H, H-27), 1.20 (s, 3H, H-25), 1.20–2.21 (m, 30H, CH and CH_2_), 2.51–3.15 (m, 16H, CH_2_NH_2_ and NHCH_2_), 3.30–3.34 (m, 1H, H-3), 5.38 (s, 1H, H-12). ^13^C NMR (MeOH, MHz): 15.9, 16.3, 17.9, 18.0, 19.5, 20.9, 24.1, 25.5, 26.1, 26.4, 26.5, 27.8, 28.6, 28.7, 31.6, 31.6, 33.5, 33.6, 33.8, 34.4, 35.1, 38.2, 38.2, 39.7, 39.8, 39.8, 40.7, 42.3, 42.9, 43.0, 47.5, 47.6, 49.8, 56.7, 79.6 (C3), 124.0 (C12), 145.2 (C13), 178.3 (C28). ESI-MS: *m*/*z* = 628 [M + H^+^].

### 2.2. General Procedure for the Synthesis of Compounds ***3***, ***4***

A mixture of methyl oleanoate (300 mg, 0.64 mmol), titanium(IV) isopropoxide (181 mg, 0.64 mmol), and spermine (130 mg, 0.64 mmol) in absolute methanol (5 mL) was stirred at room temperature for 12 h. Sodium borohydride (49 mg, 1.28 mmol) was then added at 0 °C, and the resulting mixture was stirred for an additional 2 h. The reaction was then quenched by adding water (1 mL). Stirring was maintained at room temperature for 20 min. After filtration over a pad of Celite washing with methanol and ethylacetate, the solvents were removed under vacuum, and the crude amine was purified by flash chromatography on silica gel using CH_2_Cl_2_/MeOH/NH_4_OH (7/3/1) as the eluent affording the expected coupling product **3** and **4** each as diastereomiric mixture in 35% and 40% yield.

Methyl *N*-(3-((4-((3-aminopropyl)amino)butyl)amino)propyl)-3-deoxy-olean-12-en-28-oate (**3**). ^1^H NMR (MeOH, MHz): 0.88 (s, 3H, H-30), 0.95 (s, 3H, H-23), 1.00 (s, 3H, H-26), 1.03 (s, 3H, H-29), 1.12 (s, 3H, H-24), 1.13 (s, 3H, H-27), 1.25 (s, 3H, H-25), 1.26–3.48 (m, 44H, CH and CH_2_), 3.51–3.97 (m, 5H, 3NH, NH_2_), 3.31 (s, 3H, OMe), 5.31 (s, 1H, H-12). ^13^C NMR (MeOH, MHz): 15.0, 15.9, 16.3, 16.6, 17.6, 19.6, 20.8, 21.4, 22.7, 24.0, 26.4, 27.0, 27.2, 28.7, 30.5, 30.8, 31.6, 31.9, 33.5, 33.6, 33.9, 34.8, 35.0, 38.1, 38.2, 39.0, 39.8, 40.6, 42.0, 42.8, 43.6, 47.1, 48.1, 52.2, 56.1, 56.7, 57.0, 79.7 (C3), 123.8 (C12), 145.4 (C13), 180.0 (C28).

Methyl *N*-(3-(4-(3-aminopropyl)piperazin-1-yl)propyl)-3-deoxy-olean-12-en-28-oate (**4**). ^1^H NMR (MeOH, MHz): 0.75 (s, 3H, H-30), 0.81 (s, 3H, H-23), 0.92 (s, 3H, H-26), 0.95 (s, 3H, H-29), 0.99 (s, 3H, H-24), 1.18 (s, 3H, H-27), 1.27 (s, 3H, H-25), 1.31–2.18 (m, 43H, CH and CH_2_), 2.81–3.70 (m, 4H, NHCH_2_, CH_2_NH_2_), 3.32 (s, 3H, OMe), 5.25 (s, 1H, H-12). ^13^C NMR (MeOH, MHz): 15.9, 16.3, 17.6, 19.5, 24.0, 24.1, 24.5, 25.3, 25.9, 26.4, 26.7, 27.8, 28.7, 28.9, 29.0, 30.2, 30.9, 31.6, 33.5, 33.6, 33.9, 34.6, 34.8, 38.2, 39.8, 40.6, 41.4, 41.7, 42.8, 42.8, 43.1, 43.2, 44.2, 47.1, 48.1, 52.2, 56.7, 79.7 (C3), 123.8 (C12), 145.0 (C13), 179.9 (C28). 

### 2.3. Synthesis of Compound ***6***

A mixture of compound **5** (246 mg, 0.5 mmol), spermine (101 mg, 0.5 mmol), formalin (37%, 5 mmol, 0.14 mL), copper iodide (1 mg, 0.005 mmol), and DMSO (2 mL) was stirred at 40 °C for 1 day. After the reaction was completed (as indicated by TLC), a solution of aqueous ammonia (30%, 5 mL) in water (5 mL) was added. The mixture was extracted with ethyl acetate (10 mL), and the solvents were evaporated under reduced pressure. The crude conjugate was purified by flash chromatography on silicagel using CH_2_Cl_2_/MeOH/NH_4_OH (7/3/1) as the eluent affording the expected coupling product **6** in 45% yield. 

*N*-(4-(3-aminopropyl)amino)butyl)amino)propyl)-but-2-yne-1-yl)-3-oxo-olean-12(13)-en-28-amide (**6**). ^1^H NMR (MeOH, MHz): 0.86 (s, 3H, H-30), 0.90 (s, 3H, H-23), 0.95 (s, 3H, H-26), 1.04 (s, 3H, H-29), 1.08 (s, 3H, H-24), 1.10 (s, 3H, H-27), 1.19 (s, 3H, H-25), 1.26–2.11 (m, 37H, CH, CH_2_, NH, NH_2_), 2.35–3.01 (m, 12H, 6CH_2_), 3.30–3.38 (m, 2H, CCH_2_N), 3.89–3.98 (m, 2H, CONHCH_2_), 5.38 (s, 1H, H-12). ^13^C NMR (MeOH, MHz): 15.9, 18.1, 20.9, 22.2, 24.3, 24.5, 24.6, 24.9, 25.0, 25.4, 25.6, 26.6, 27.2, 28.7, 30.1, 31.8, 33.5, 33.7, 34.4, 35.3, 36.3, 38.1, 40.4, 40.9, 41.5, 42.7, 43.2, 43.9, 44.5, 45.3, 47.7, 48.4, 48.7, 52.2, 53.0, 54.9, 55.6, 56.7, 74.7, 83.2, 124.0 (C12), 145.5 (C13), 172.5 (C28), 218.2 (C3). ESI-MS: *m*/*z* = 706 [M + H^+^].

### 2.4. Bacterial Strains

The antimicrobial properties of oleanane polyamino-derivatives were tested against Gram-positive bacteria (*Bacillus cereus* spp. (**BC**), *Streptococcus faecalis* (**SF**), *S. aureus* (**SA**) ATCC 25923 and CIP 7625) and Gram-negative bacteria (*Klebsiella aerogenes* (**KA**) EA 289, *Escherichia coli* (**EC**) ATCC 28922 and CIP 54.8, and *Pseudomonas aeruginosa* (**PA**)ATCC 27853 and 100720, and *Salmonella enterica* spp. (**SA**)). Colistine purchased from Sigma (Saint-Quentin-Fallavier, France) and erythromycine from Amdipharm Ltd. (Dublin, Ireland), were chosen as positive controls against Gram-negative and Gram-positive bacteria, respectively.

### 2.5. In Vitro Antibacterial Screening

All experiments were performed on the Bac-Screen platform (UMR-MD1, Marseille, France), using a robotic Freedom EVO 150 liquid handling system (Tecan Lyon, France). Doxycycline, polymyxin-B nonapeptide (PMBn) and polymyxin-B (PMB) were purchased from Sigma; erythromycin-lactobionate was purchased from Amdipharm Ltd. They were dissolved in ethanol or water, as further precised. Nitrocefin was purchased from Oxoid (Basingstoke, UK) and dissolved in DMSO 5%.

### 2.6. Antibiotic Susceptibility Testing

Susceptibilities of bacteria to oleanane polyamino-derivatives were determined in microplates by the standard broth dilution method in accordance with the recommendations of the Comité de l’Antibiogramme de la Société Française de Microbiologie (CA-SFM) [28]. The stock solutions of hychloride salt of the derivatives were freshly prepared for each experiment in water. Briefly, the Minimal Inhibitory Concentrations (MICs) were determined starting with an inoculum of 10^5^ CFU in 200 µL of MH broth containing twofold serial dilutions of oleanane polyamino-derivatives ranging from 128 µg/mL to 0.25 µg/mL. The MIC was defined as the lowest concentration of drug that completely inhibited visible growth after incubation for 18 h at 37 °C. All MIC determinations were repeated in triplicate in independent experiments.

### 2.7. Determination of MICs of Doxycycline or Erythromycine in the Presence of Synergizing Compounds

The effects of combinations of each compound and doxycycline or erythromycin was evaluated in sterile 96-well microplates. Doxycycline and erythromycin concentration was fixed at 2 µg/mL. to determine the lowest concentration of oleanane polyamino-derivatives acting as an adjuvant capable of decreasing the MIC of doxycycline and erythromycin at this sensitivity threshold. (Clinical and Laboratory Standards Institute) [29], The bacterial concentration was adjusted to 1.5 × 10^5^ CFU/well. The MIC for each drug combination was determined after 18 h of incubation at 37 °C. All MIC determinations were repeated at least three times in independent experiments.

### 2.8. Outer Membrane Permeation Assay

An overnight culture of *P. aeruginosa* PA01 was diluted 100-fold into 10 mL of MHII broth containing 10 mg/mL of imipenem to induce a high level of β-lactamases. After bacteria grown until the absorbance at 600 nm (OD600) reached 0.5, cells were recovered by centrifugation (4000× *g* for 20 min at 20 °C) and washed twice with sterile 20 mM potassium phosphate buffer (pH 7.2) supplemented with 1 mM MgCl_2_. The pellets were recovered and the OD600 was adjusted to 0.5 with the buffer without shaking to avoid the cells bursting. Each compound was tested at a 128 µg/mL concentration. Three control wells were used: a negative control containing the PPB buffer, two positive controls: the first containing the PMB and the second containing the PMBn. One hundred microliters of the bacterial suspension were added to all wells previously filled, then 50 μL of nitrocefin was added at a final concentration of 50 μg/mL. Plates were read at 490 nm using an Infinite M200 microplate reader (Tecan) equipped with a spectrophotometer for 1 h with a 1-min interval between two measurements. Experiments were performed in triplicate.

## 3. Results

### 3.1. Chemistry

The synthesis of a series of polyamine conjugates of oleanane type was performed by using the 3-oxo-oleanolic acid **1** as starting material and which is easily obtained from Jones oxidation of oleanolic acid [30]. Different types of diamines, natural (spermine, cadaverine) and other commercially available polyamines, containing different amount of methylene groups, as well as the cyclic moieties (such as morpholine, piperazine, pyrrolidone), etc. were used to obtain structure-activity relationships data (SAR).

**Scheme 1:**
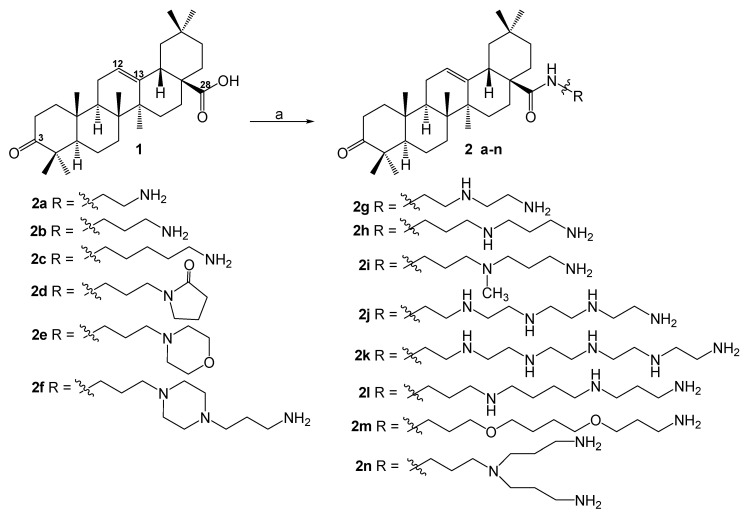
Synthesis of olenonic diamine and polyamine conjugates **2a**–**2n**. Reagents and conditions: R-NH_2_, BOP, DIPEA, DCM, 20 °C, 12 h.

Subsequent reaction of compound **1** with the corresponding diamines or polyamines using BOP coupling reagent [31] lead to the formation of the expected oleanane derivatives **2a**–**2n** in yields ranging from 49 to 80% (Scheme 1). 

**Scheme 2:**
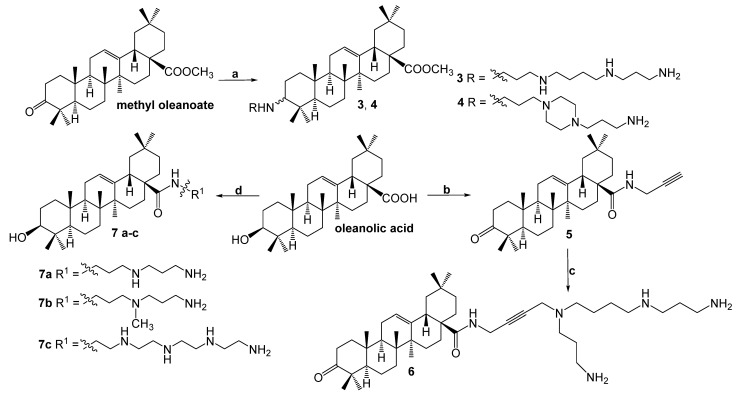
Synthesis of C3 and C28 oleanolic acid polyamine conjugates. Reagents and conditions: a. i. R-NH_2_, Ti(O*i*-Pr)_4_, MeOH, 20 °C, 12 h; ii. NaBH_4_, −78 °C, 2 h. b. i. CrO_3_, H_2_SO_4_, 20 °C, 2 h; ii. (COCl)_2_, CH_2_Cl_2_, 20 °C, 2 h; iii. NH_2_CH_2_CCH, Et_3_N, CH_2_Cl_2_, 20 °C, 5 h. c. spermine, formalin, CuI (cat.), DMSO, 40 °C, 24 h. d. R-NH_2_, BOP, DIPEA, DCM, 20 °C, 12 h.

In order to obtain structure-activity relationships data (SAR) some other derivatives were synthesized (Scheme 2) such as amides of oleanonic acid **7a**–**7c** obtained under the same conditions than previously in 47–60% yield. On the other hand, by using an efficient titanium-reductive amination developed in our laboratory [32,33] the 3-*N*-polyamino-3-deoxy-oleanane derivatives **3**, **4** each as a diastereomeric mixture from methyl oleanoate with not optimized yields (35–40%) were also prepared. Furthermore, Mannich reaction of *N*-propargylamide of oleanonic acid **5** with spermine and formalin in DMSO in the presence of catalytic amounts of CuI afforded the Mannich base **6** in 45% yield [34].

The structure of all the compounds was confirmed by NMR spectroscopy. Typically, for compounds **2a**–**n**, **7a**–**c** the signals of the amide function at δ 172–180 ppm (^13^C NMR), as well as the signals of methylene groups of polyamine functions as multiplets at δ 1.29–4.00 ppm were characteristic and well assigned. In the case of compound **6** the absence of signals of acetylene fragments (at δ 71.6–80.0 ppm (^13^C NMR) and δ 2.21–2.23 ppm (^1^H NMR)) and presence of methylene groups of spermine moiety as multiplets at δ 2.35–3.98 ppm confirmed its formation unambiguously.

### 3.2. Antibacterial Activities 

All the previously synthesized compounds were evaluated for their antimicrobial activities against both Gram-positive and Gram-negative bacterial strains (Table 1). Thus, except for derivatives **2d**, **2e**, **2k**, **3** and **4**, all the compounds presented good to moderate activity against Gram-positive *S. aureus,* *S. faecalis* and *B. cereus* bacteria varying from 3.125 to 200 µg/mL. Nevertheless, compounds **2a**–**2c** are only efficient against Gram-positive bacteria, while all other compounds possess important antimicrobial activities against Gram-negative *E. coli*, *P. aeruginosa*, *S. enterica*, and EA289 bacteria with MICs ranging from 6.25 to 200 µg/mL. More interestingly, compounds **2i** and **6** appeared possessing low MICs against various multidrug resistant bacteria such as *P. aeruginosa* CIP100720 and *K. aerogenes* EA289.

For a second time, our compounds were then tested for their ability to restore the antibiotic activity of doxycycline and erythromycin at a 2 µg/mL concentration in a synergistic assay against *P. aeruginosa* and *E. coli*, respectively (Table 2). The compounds were used at a sub-inhibitory concentration corresponding to their respective MICs to avoid an intrinsic action and to ascertain that the effect observed resulted from the combination of the molecules used. Thus, only derivative **6** lead to a moderate improvement in terms of antimicrobial activities of the different selected combinations against both *P. aeruginosa* and *E. coli*.

**Figure 1:**
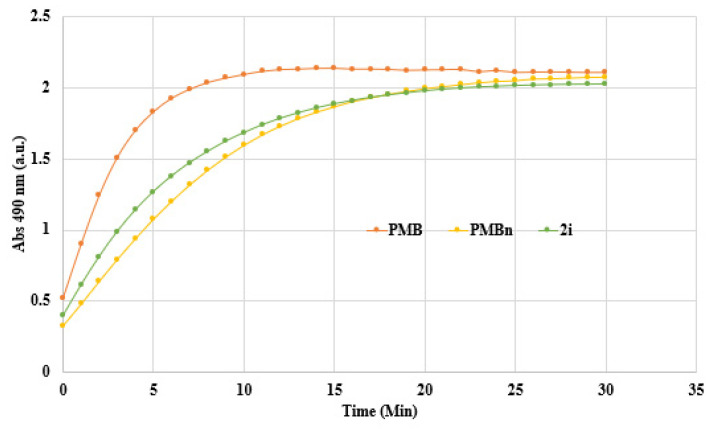
Nitrocefin hydrolysis kinetics in presence of compounds **2i** at 18 µg/mL and compared to PMB and PMBn.

To study the mechanism of action of the best compound **2i**, its effect on the integrity of the outer membrane was evaluated on the well-known *P. aeruginosa* PA01 strain. The method used is based on the measurement of the kinetic of the chromogenic β-lactam nitrocefin by periplasmic beta-lactamases causing a change in color from yellow to red and subsequently permitting to relate it to the integrity of the outer membrane of the bacteria [35,36,37]. The curves of nitrocefin hydrolysis kinetic obtained in the presence of derivative **2i** and in the presence of the two positive controls polymyxin B PMB and polymyxin B nona (PMBn) known for their permeabilizing effect of the external bacterial membrane are reported in Figure 1. At the same concentration of 128 μM, the results showed a rapid disruption of the bacterial membrane by the derivative **2i** as for positive control PMBn. It is noteworthy that these results are in good correlation with those obtained in our previous antibiotic-adjuvant test (Table 2).

Finally, pharmacokinetic and toxicological properties of the lead compound **2i** were assessed computationally using a consensus approach. The choice of services was based on applicability criterion since relevant results can only be obtained for compounds in the training domain. As shown in Table 3, all approaches predicted poor aqueous solubility with an average of 4 µM. Additionally, the consensus is evident for high logP value, which reflects that **2i** is highly lipophilic and, therefore, is likely to be cell-permeable and show a moderate to high metabolic rate. Accordingly, **2i** is predicted to be suitable for oral administration with expected bioavailability greater than 30% (ADMETlab estimation). However, after absorption the compound is anticipated to be substantially bound to plasma proteins. The volume of distribution (V_D_), the apparent volume into which the drug is distributed at equilibrium and before clearance begins, is predicted to be low.

## 4. Discussion

This study was conducted on different bacterial strains including Gram-negative bacteria that have been proved to be the less sensitive strains than the Gram-positive ones, being resistant to many classes of antibiotics due to their outer lipidic membrane that restricts the access of molecules to the periplasm. Recently, we reported a great activity of polyaminosterol derivatives for their intrinsic antimicrobial activities [35] or for their use in combination [42]. Concerning the mechanism of action of our best derivative **2i** (derived from *N*-methyl-norspermidine), our data suggest that this compound disrupts the outer membrane integrity of the *P. aeruginosa* Gram-negative bacteria by acting in a similar manner to PMBn. It is well admitted that disruption of Gram-negative bacterial cytoplasmic membrane constituted the main mechanism of action by polymyxin B [43]. Conversely, polymyxin B nonapeptide (PMBN) that lacks the fatty acyl tail does not possess activity but is able to compromise the outer membrane integrity [44,45,46,47]. Thus, an outer membrane permeabilization could occur because of the interactions between charges but the hydrophobic acyl tail could improve this phenomenon. The lack of activity of PMBN as well as our derivative **2i**, however, tends to indicate that the outer membrane is a site of interaction, but it is not the killing target.

All of our data clearly suggest that the nature of the polyamine fragment, as well as the structure of the oleanane core plays an important role in the potential activities of tested compounds. Polyamine amides with 3β-hydroxy-group **7a** and **7b** did not show high activity, at the same time oxidation to the 3-oxo-group in case of analogs **2h** and **2i** was effective. However, 3β-hydroxy-derivative **7c** showed higher activity against Gram-negative *S. enterica* (MIC 12.5 µg/mL) and EA289 (MIC 25 µg/mL) and against Gram-positive *B. cereus* (MIC 25 µg/mL) in comparison with 3-oxo-analog **2j**. Diaminoderivatives **2a**, **2b** and **2c** possess antimicrobial activity only against Gram-positive bacteria with MICs from 6.25 to 200 µg/mL. Among the 3-oxo-oleanane-polymethylenpolyamine amides only pentaamine **2j** and spermine **2l** derivatives presented antimicrobial activity against all the considered strains whereas aminopropoxy **2m** analog of **2j** did not lead to any activity against Gram-negative bacteria. Additionally, product **3** and *N-*propargyl amide of 3-oxo-oleanolic acid **5** were not active, while modification of the latter one in the Mannich base **6** with spermine fragment led to a high activity against all the bacterial strains. Among the derivatives possessing heterocyclic moieties such as 2-oxopyrrolidino- **2d** and morpholino- **2e** analogs they appeared not active, while piperazine containing derivative **2f** possess antimicrobial activities with the highest value of MICs of 50 and 12.5 µg/mL, against Gram-negative *E. coli* ATCC 28922 and Gram-positive *S. aureus*, respectively. It is also noteworthy that methyl ester derivative **4** presenting a piperazine fragment at C3 was not active.

Increasing methylene group amounts in a series of triamine derivatives provided a positive effect: thus, compounds **2h** and **2i** possess higher antimicrobial activity than previously described for **2g** [26]. Substituted secondary amine group with methyl fragment (compound **2i**), provided increasing of activity compared with **2h**. Additionally, derivatives **2h** and **2i** presenting a 3β-hydroxy group (**7a**, **7b**) did not show activity. 

On the other hand, the ADME study means that most of the administered **2i** will be confined to the circulatory system. Generally, it indicates a low therapeutic index and necessitates a high loading dose to achieve therapeutic plasma concentration. High lipophilicity is known to contribute to human plasma protein binding the most [48]. In particular, α_1_-acid glycoprotein primarily binds basic and hydrophobic compounds, e.g., steroids [49], which renders it a suitable carrier for **2i**. Blood-brain permeation for **2i** cannot be ruled out unambiguously. All services predicted that it will be oxidized by cytochrome P450 3A4. The compound is unlikely to inhibit common cytochromes but CYP3A4 induction may affect the pharmacokinetic profiles of concomitantly administered CYP3A4-metabolized drugs [50]. Clearance is predicted to be relatively slow, however, due to low V_D_ and high plasma protein binding elimination half-life T_1/2_ is also short. According to predicted moderate oral acute toxicity the compound can be attributed to Category 3 according to GHS classification with no toxicity towards heart, liver, mutagenic or carcinogenic properties.

Overall, computational ADMET profiling renders compound **2i** as a suitable starting point for pharmacokinetic optimization. It is orally available and has favorable safety properties. Possible drawbacks include low solubility, volume of distribution and short plasma half-life. These issues might be addressed by lipophilicity management maintaining metabolic stability (e.g., introduction of unsaturated carbon-carbon bonds [51] and replacement of hydrogen with fluoride [52]).

## 5. Conclusions

The series of 21 oleanolic acid derivatives containing di- and polyamine fragments at position C3 and C28 was synthesized and evaluated for their antimicrobial activities against both Gram-positive and Gram-negative bacterial. Almost all series presented good to moderate Minimum Inhibitory Concentrations (MIC) against Gram-positive *S. aureus*, *S. faecalis* and *B. cereus* bacteria, moreover compounds possess important antimicrobial activities against Gram-negative *E. coli*, *P. aeruginosa*, *S. enterica*, and *EA289* bacteria with MICs ranging from 6.25 to 200 µg/mL. The SAR data showed that the nature of the polyamine fragment, as well as differences in the structure of oleanolic acid plays an important role in the potential activities of tested compounds. The testing of the ability to restore the antibiotic activity of doxycycline and erythromycin at a 2 µg/mL concentration in a synergistic assay showed that only Mannich base with spermine fragment **6** lead to a moderate improvement in terms of antimicrobial activities of the different selected combinations against both *P. aeruginosa* and *E. coli*. The study of the mechanism of action of the most important compound in this series (amide **2i** derived from *N*-methyl-norspermidine) showed the effect of disruption of the outer bacterial membrane of *P. aeruginosa* PA01 cells. Computational ADMET profiling renders compound **2i** as a suitable starting point for pharmacokinetic optimization.

## Figures and Tables

**Table 1:** Antimicrobial activities of oleanolic acid derivatives **2a**–**n**, **3**–**6**, **7a**–**c**.

Compound	Minimum Inhibitory Concentration ^a^ (µM)									
	Gram-Negative Bacteria ^b^						Gram-Positive Bacteria ^c^			
	PA ATCC 27853	PA CIP 100720	EC ATCC 28922	EC CIP 54.8	SE	EA289	SA ATCC 25923	SA CIP 76.25	SF sp.	BC sp.
**2a**	>200	>200	200	200	50	200	6.25	12.5	25	12.5
**2b**	>200	>200	200	>200	200	200	12.5	25	12.5	12.5
**2c**	>200	>200	100	>200	100	100	12.5	25	6.25	12.5
**2d**	>200	>200	>200	>200	NT	NT	>200	>200	200	NT
**2e**	>200	>200	>200	>200	>200	>200	>200	>200	>200	>200
**2f**	50	200	50	200	25	25	12.5	25	12.5	12.5
**2g**	50	100	25	50	25	50	12.5	12.5	12.5	12.5
**2h**	50	100	25	50	12.5	25	12.5	25	12.5	12.5
**2i**	25	100	25	50	12.5	12.5	6.25	12.5	6.25	6.25
**2j**	200	>200	50	50	25	25	12.5	NT	12.5	12.5
**2k**	>200	>200	>200	>200	NT	NT	>200	>200	>200	NT
**2l**	>200	>200	25	50	NT	NT	3.125	6.25	200	NT
**2m**	>200	>200	>200	>200	NT	NT	100	NT	50	NT
**2n**	200	>200	100	100	NT	NT	100	NT	200	NT
**3**	>200	>200	>200	>200	NT	NT	>200	NT	>200	NT
**4**	>200	>200	>200	>200	>200	>200	>200	>200	>200	>200
**5**	>200	>200	>200	>200	NT	NT	>200	>200	>200	NT
**6**	12.5	25	6.25	12.5	12.5	25	12.5	NT	6.25	NT
**7a**	200	>200	100	200	25	50	25	50	100	25
**7b**	>200	>200	100	100	50	100	50	100	50	25
**7c**	100	>200	25	50	12.5	25	12.5	25	NT	25

^a^ Values are mean of triplicate determination. ^b^ PA (*P. aeruginosa*), EC (*E. coli*), SE (*S. enterica*), EA289 (*K. aerogenes*). ^c^ SA (*S. aureus*), SF (*S. faecalis*), BC (*B. cereus*).

**Table 2:** Concentration (μg/mL) of compounds **2a**–**n**, **3**–**6**, **7a**–**c** used to restore doxycycline and eryhtromycin activity (used at 2 µg/mL) against *P. aeruginosa* ATCC 27853 and *E. coli* ATCC 28922, respectively.

Cpd/Strains		2a	2b	2c	2d	2e	2f	2g	2h	2i	2j	2k	2l	2m	2n	3	4	5	6	7a	7b	7c
MIC (µg/mL) ^c^	*P. aeruginosa* ATCC 27853 ^a^	200	200	200	>200	>200	100	25	50	25	100	>200	100	>200	200	>200	>200	>200	6.25	200	200	100
	*E. coli* ATCC 28922 ^b^	100	200	200	>200	>200	100	50	25	25	25	>200	50	>200	50	>200	>200	>200	3.125	50	200	12.5

^a^ Concentration of the selected compound necessary to restore doxycycline activity used at 2 µg/mL. ^b^ Concentration of the selected compound necessary to restore erythromycin activity used at 2 µg/mL. ^c^ Values are mean of triplicate determination.

**Table 3:** Predicted ADMET characteristics of compound **2i**.

Category	Property	Predicted Values				Consensus Value ^1^
		ADMET 1ab [38]	ADMET Lab 2.0 [39]	Admet SAR [40]	SwissADME [41]	
Physicochemical	Water solubility (l g mol/L)	−5.77	−4.65	−3.19	−7.99	−5.40
	LogP	7	5.285	7	6.18	6.37
Absorption	Intestinal absorption (human, %)	+	+	+	Low	+
	Human oral bioavailability	+	+	+		+
	P-glycoprotein substrate	-	-	+	-	-
	P-glycoprotein I inhibitor	+	-	+		+
Distribution	V_D_ (human, L/kg)	0.836	0.876			0.856
	Plasma protein binding (%)	81.68	59.69	100		90.84
	BBB permeability	+	-	+	-	-
Metabolism	CYP1A2 inhibitior	-	-	-	-	-
	CYP2C19 inhibitior	-	-	-	-	-
	CYP2C9 inhibitior	-	-	-	-	-
	CYP2D6 inhibitior	-	+	-	-	-
	CYP2D6 substrate	-	+	-		-
	CYP3A4 inhibitior	-	+	-	-	-
	CYP3A4 substrate	+	+	+		+
Excretion	Total clearance (mL/min/kg)	1.24	5.47			3.36
	T_1/2_ (h)	1.93	0.175			1.05
Toxicity	AMES toxicity	-	-	-		-
	hERG I inhibitor	-	-			-
	hERG II inhibitor	-	-			-
	Acute oral toxicity (LD_50_, mg/kg)	279.17		368.13		323.65
	Hepatotoxicity	-	-	-		-
	Skin sensitization	-	-			-
	Carcinogenicity		-	-		-

^1^ Mean of numerical values or consensus of categorical values, if possible.

## Data Availability

Not applicable.

## Supplementary Materials

The following are available online at https://www.mdpi.com/article/10.3390/antibiotics11010094/s1. Table S1: SMILES Code of the synthesized compounds, Figures S1–S40: ^1^H and ^13^C NMR spectra of all the synthesized compounds.
