# Supplementary material for: Development of New Antimicrobial Oleanonic Acid Polyamine Conjugates
Source: Antibiotics (Basel). 2022 Jan 12;11(1):94. doi: 10.3390/antibiotics11010094 (PMC8772916; doi:10.3390/antibiotics11010094)
Supplement: Supplementary file 1 [file antibiotics-11-00094-s001.zip › antibiotics-1540058-supplementary.pdf]

**SUPPORTING INFORMATION**

# Development of new antimicrobial oleanonic acid polyamine conjugates

**Elmira F. Khusnutdinova[1,2*], Véronique Sinou[2], Denis A. Babkov[3], Oxana Kazakova[1] and Jean Michel Brunel[2*]**

[1] Ufa Institute of Chemistry UFRC RAS, 71, pr. Oktyabrya, 450054 Ufa, Russian Federation.
[2]Aix Marseille Univ, INSERM, SSA, MCT, 13385 Marseille, France.
[3]Scientific Center for Innovative Drugs, Volgograd State Medical University, Novorossiyskaya st. 39, 400087 Volgograd, Russian Federation.

* Correspondence: EFK ElmaH@inbox.ru, JMB bruneljm@yahoo.fr

| Cpd | SMILES |
|---|---|
| **2a** | CC1(C)CC[C@]2(C(NCCN)=O)CC[C@@]3(C)[C@]4(C)CC[C@@]5([H])C(C)(C)C(CC[C@]5(C)[C@@]4([H])CC=C3[C@]2([H])C1)=O |
| **2b** | CC1(C)CC[C@]2(C(NCCCN)=O)CC[C@@]3(C)[C@]4(C)CC[C@@]5([H])C(C)(C)C(CC[C@]5(C)[C@@]4([H])CC=C3[C@]2([H])C1)=O |
| **2c** | CC1(C)CC[C@]2(C(NCCCCCN)=O)CC[C@@]3(C)[C@]4(C)CC[C@@]5([H])C(C)(C)C(CC[C@]5(C)[C@@]4([H])CC=C3[C@]2([H])C1)=O |
| **2d** | CC1(C)CC[C@]2(C(NCCCN3CCCC3=O)=O)CC[C@@]4(C)[C@]5(C)CC[C@@]6([H])C(C)(C)C(CC[C@]6(C)[C@@]5([H])CC=C4[C@]2([H])C1)=O |
| **2e** | CC1(C)CC[C@]2(C(NCCCN3CCOCC3)=O)CC[C@@]4(C)[C@]5(C)CC[C@@]6([H])C(C)(C)C(CC[C@]6(C)[C@@]5([H])CC=C4[C@]2([H])C1)=O |
| **2f** | CC1(C)CC[C@]2(C(NCCCN3CCN(CCCN)CC3)=O)CC[C@@]4(C)[C@]5(C)CC[C@@]6([H])C(C)(C)C(CC[C@]6(C)[C@@]5([H])CC=C4[C@]2([H])C1)=O |
| **2g** | CC1(C)CC[C@]2(C(NCCNCCN)=O)CC[C@@]3(C)[C@]4(C)CC[C@@]5([H])C(C)(C)C(CC[C@]5(C)[C@@]4([H])CC=C3[C@]2([H])C1)=O |
| **2h** | CC1(C)CC[C@]2(C(NCCCNCCCN)=O)CC[C@@]3(C)[C@]4(C)CC[C@@]5([H])C(C)(C)C(CC[C@]5(C)[C@@]4([H])CC=C3[C@]2([H])C1)=O |
| **2i** | CC1(C)CC[C@]2(C(NCCCN(C)CCCN)=O)CC[C@@]3(C)[C@]4(C)CC[C@@]5([H])C(C)(C)C(CC[C@]5(C)[C@@]4([H])CC=C3[C@]2([H])C1)=O |
| **2j** | CC1(C)CC[C@]2(C(NCCNCCNCCNCCN)=O)CC[C@@]3(C)[C@]4(C)CC[C@@]5([H])C(C)(C)C(CC[C@]5(C)[C@@]4([H])CC=C3[C@]2([H])C1)=O |
| **2k** | CC1(C)CC[C@]2(C(NCCNCCNCCNCCNCCN)=O)CC[C@@]3(C)[C@]4(C)CC[C@@]5([H])C(C)(C)C(CC[C@]5(C)[C@@]4([H])CC=C3[C@]2([H])C1)=O |
| **2l** | CC1(C)CC[C@]2(C(NCCCNCCCCNCCCN)=O)CC[C@@]3(C)[C@]4(C)CC[C@@]5([H])C(C)(C)C(CC[C@]5(C)[C@@]4([H])CC=C3[C@]2([H])C1)=O |

| | |
|---|---|
| **2m** | CC1(C)CC[C@]2(C(NCCCOCCCCOCCCN)=O)CC[C@@]3(C)[C@]4(C)CC[C@@]5([H])C(C)(C)C(CC[C@]5(C)[C@@]4([H])CC=C3[C@]2([H])C1)=O |
| **2n** | CC1(C)CC[C@]2(C(NCCCN(CCCN)CCCN)=O)CC[C@@]3(C)[C@]4(C)CC[C@@]5([H])C(C)(C)C(CC[C@]5(C)[C@@]4([H])CC=C3[C@]2([H])C1)=O |
| **3** | CC1(C)C(NCCCNCCCCNCCCN)CC[C@@]2(C)[C@@]1([H])CC[C@]3(C)[C@]2([H])CC=C4[C@@]3(C)CC[C@]5(C(OC)=O)[C@@]4([H])CC(C)(C)CC5 |
| **4** | CC1(C)C(NCCCN(CC2)CCN2CCCN)CC[C@@]3(C)[C@@]1([H])CC[C@]4(C)[C@]3([H])CC=C5[C@@]4(C)CC[C@]6(C(OC)=O)[C@@]5([H])CC(C)(C)CC6 |
| **5** | CC1(C)C(CC[C@@]2(C)[C@@]1([H])CC[C@]3(C)[C@]2([H])CC=C4[C@@]3(C)CC[C@]5(C(NCC#C)=O)[C@@]4([H])CC(C)(C)CC5)=O |
| **6** | CC1(C)C(CC[C@@]2(C)[C@@]1([H])CC[C@]3(C)[C@]2([H])CC=C4[C@@]3(C)CC[C@]5(C(NCC#CCN(CCCN)CCCCNCCCN)=O)[C@@]4([H])CC(C)(C)CC5)=O |
| **7a** | CC1(C)[C@@H](O)CC[C@@]2(C)[C@@]1([H])CC[C@]3(C)[C@]2([H])CC=C4[C@@]3(C)CC[C@]5(C(NCCCNCCCN)=O)[C@@]4([H])CC(C)(C)CC5 |
| **7b** | CC1(C)[C@@H](O)CC[C@@]2(C)[C@@]1([H])CC[C@]3(C)[C@]2([H])CC=C4[C@@]3(C)CC[C@]5(C(NCCCN(C)CCCN)=O)[C@@]4([H])CC(C)(C)CC5 |
| **7c** | CC1(C)[C@@H](O)CC[C@@]2(C)[C@@]1([H])CC[C@]3(C)[C@]2([H])CC=C4[C@@]3(C)CC[C@]5(C(NCCNCCNCCNCCN)=O)[C@@]4([H])CC(C)(C)CC5 |

**Table S1:** SMILES Code of the synthesized compounds

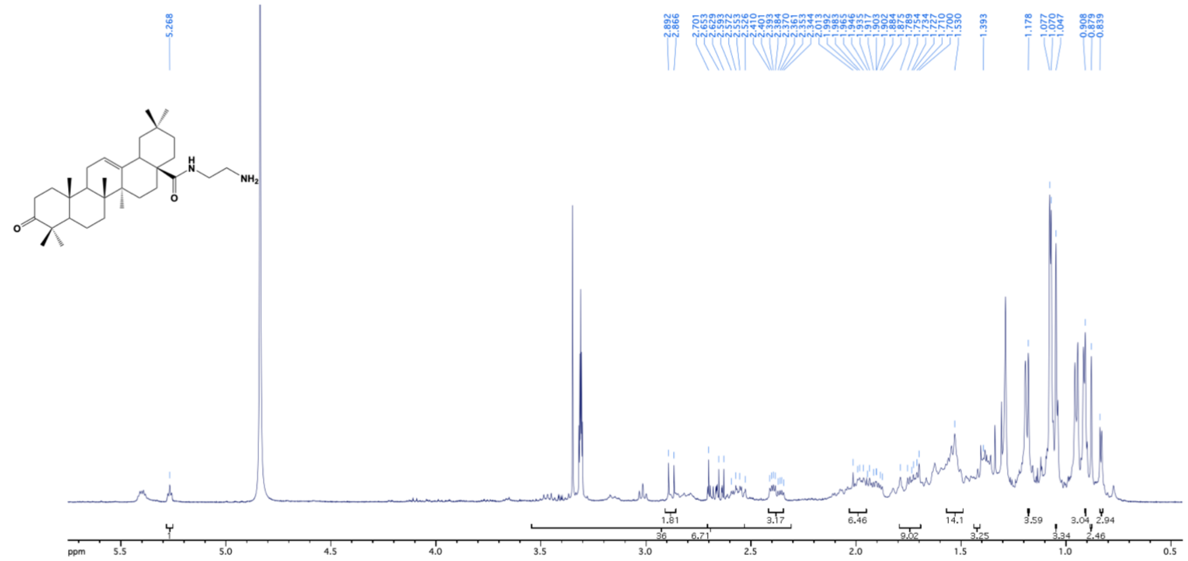


Figure S1. $^{1}$H NMR spectrum of compound **2a**

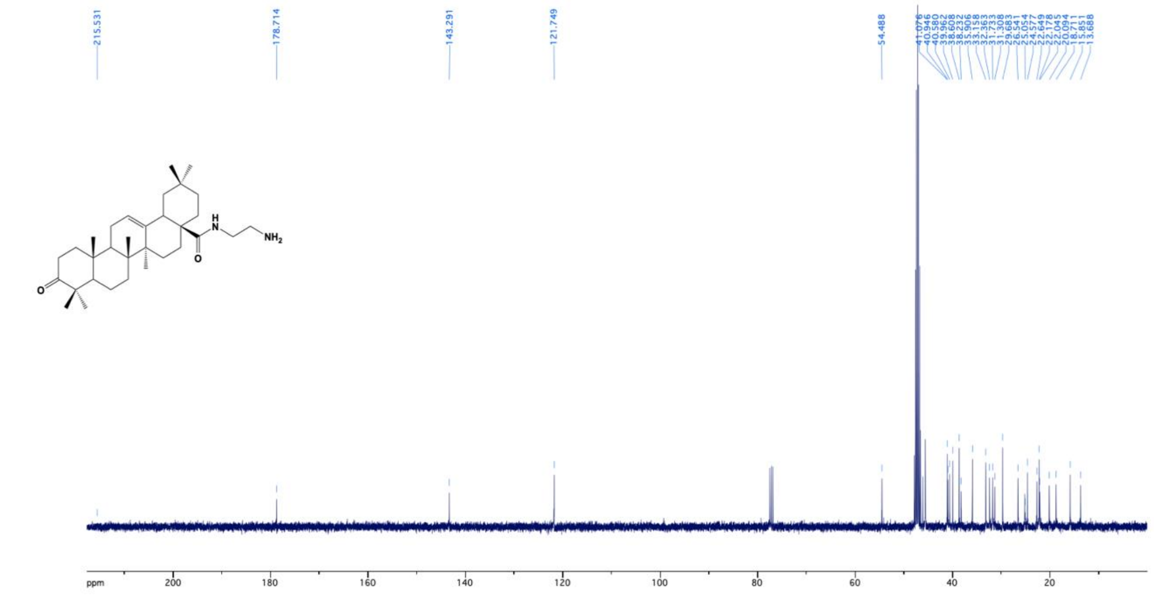


Figure S2. $^{13}$C NMR spectrum of compound **2a**

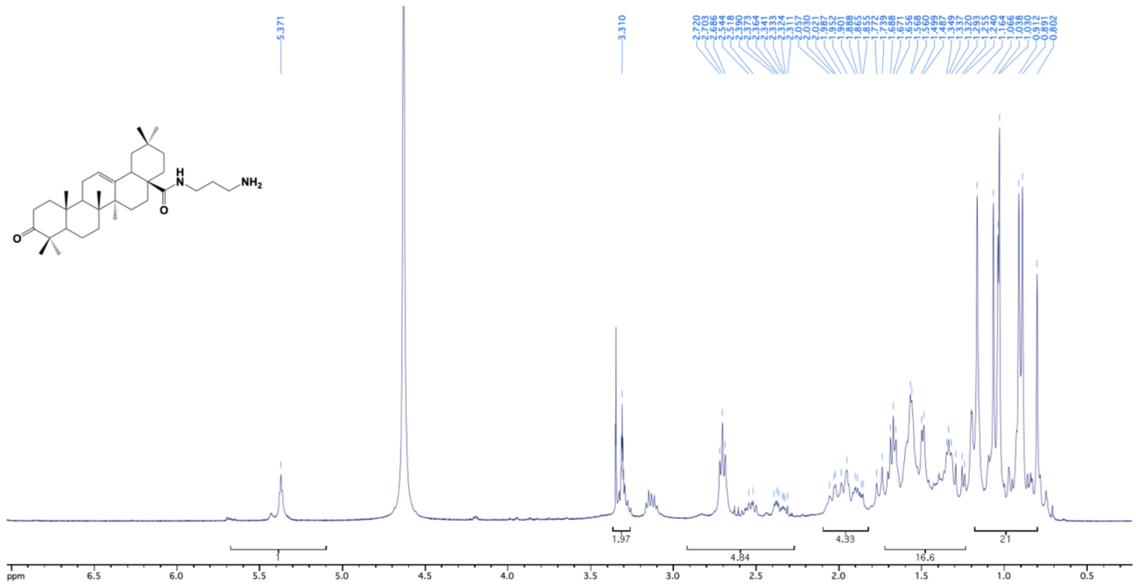


Figure S3. $^{1}$H NMR spectrum of compound **2b**

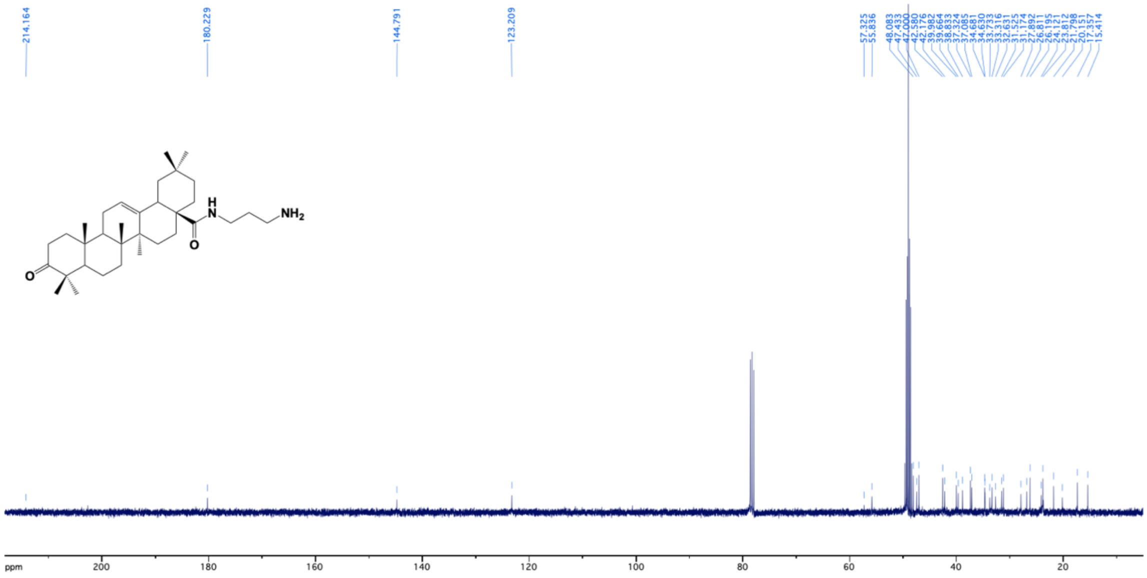


Figure S4. $^{13}$C NMR spectrum of compound **2b**

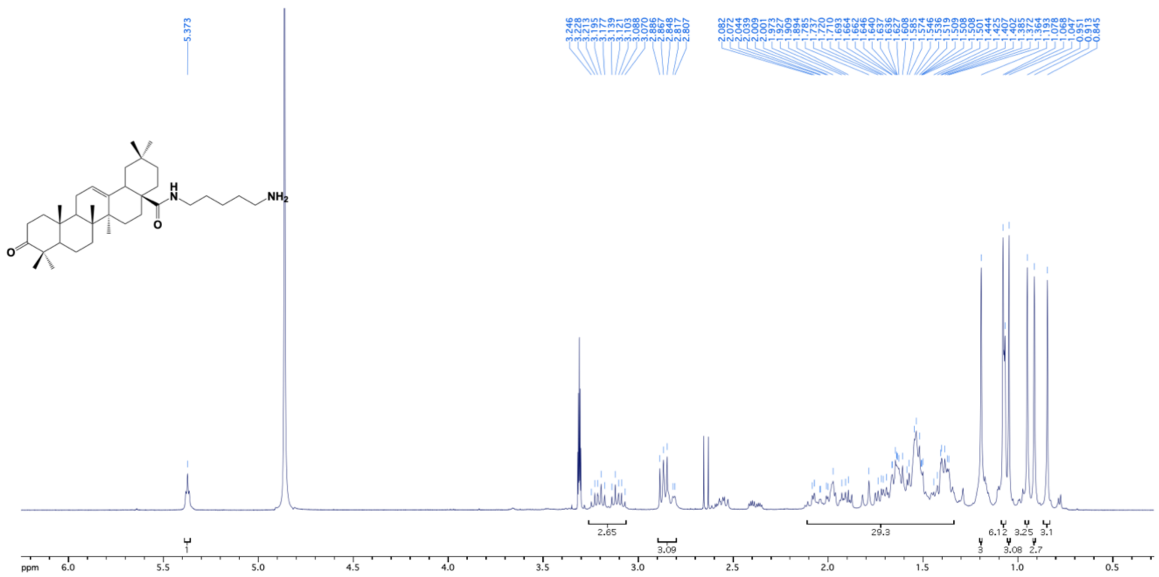

Figure S5. $^{1}$H NMR spectrum of compound **2c**

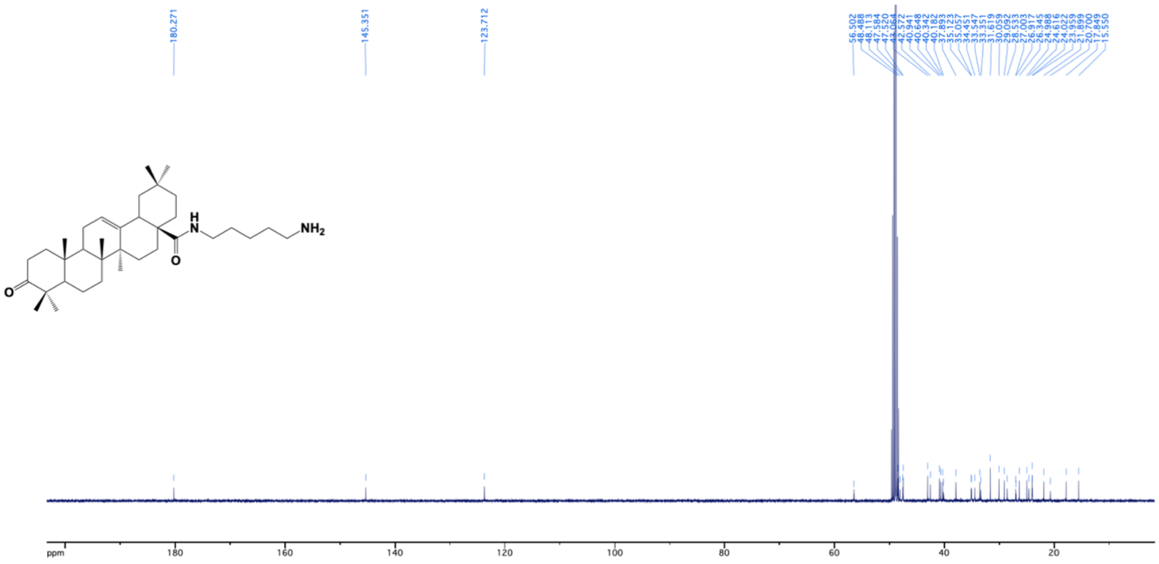

Figure S6. $^{13}$C NMR spectrum of compound **2c**

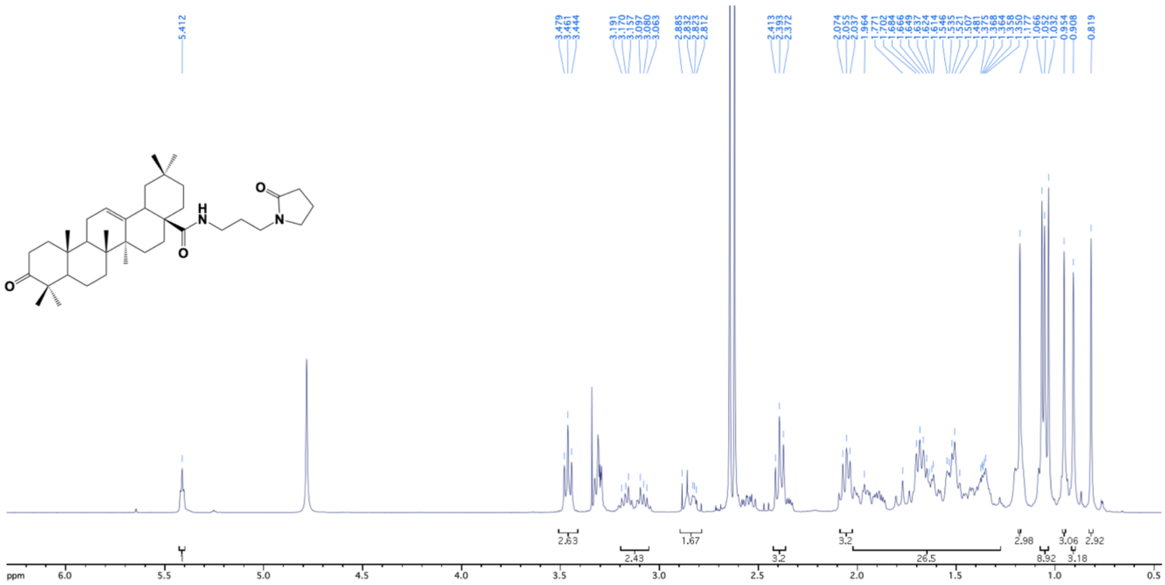

Figure S7. $^{1}$H NMR spectrum of compound **2d**

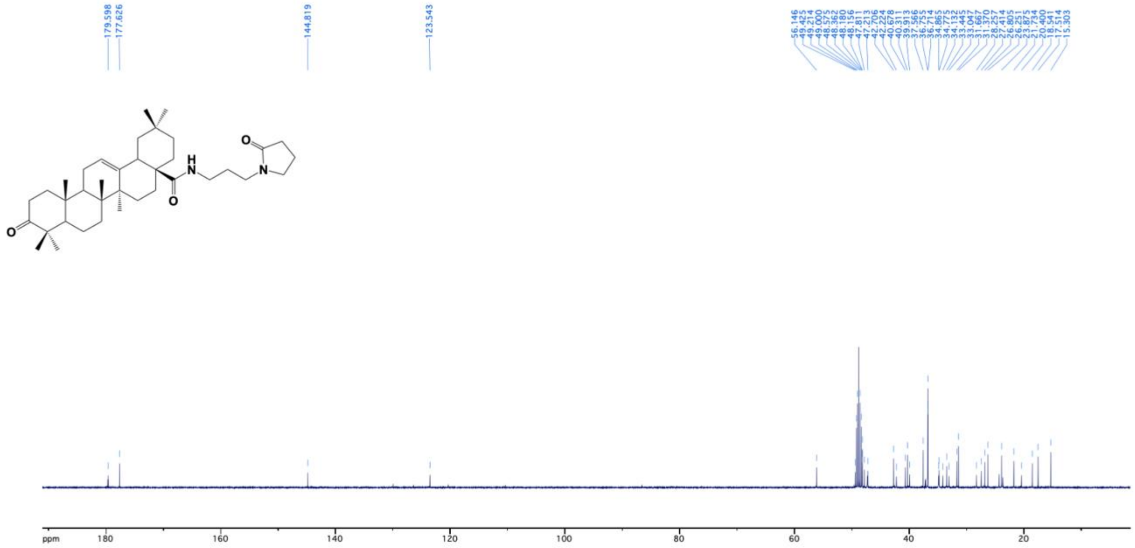

Figure S8. $^{13}$C NMR spectrum of compound **2d**

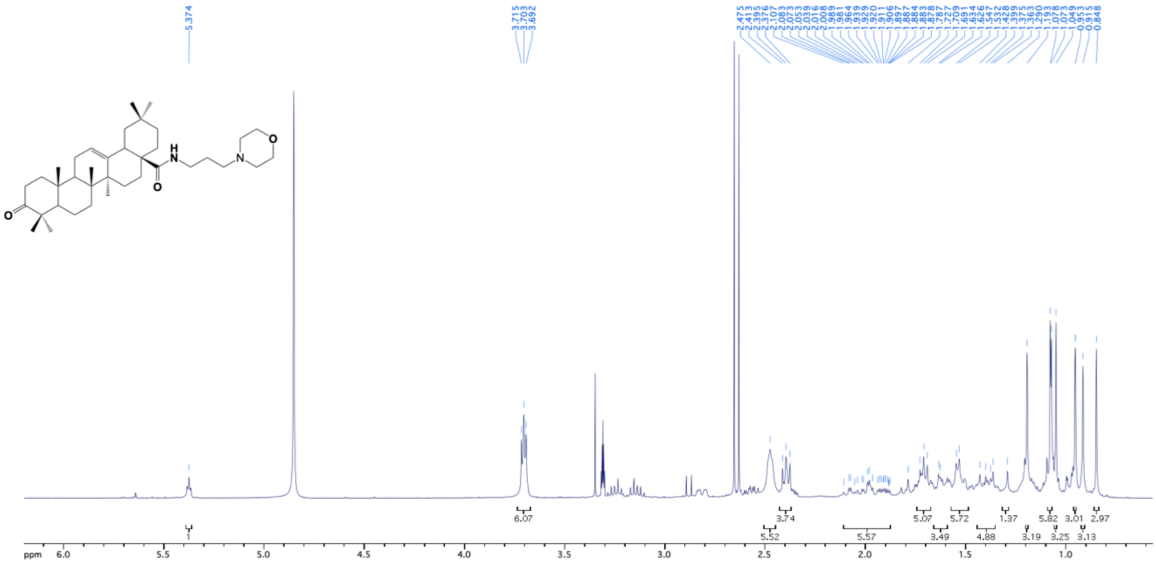

Figure S9. $^{1}$H NMR spectrum of compound **2e**

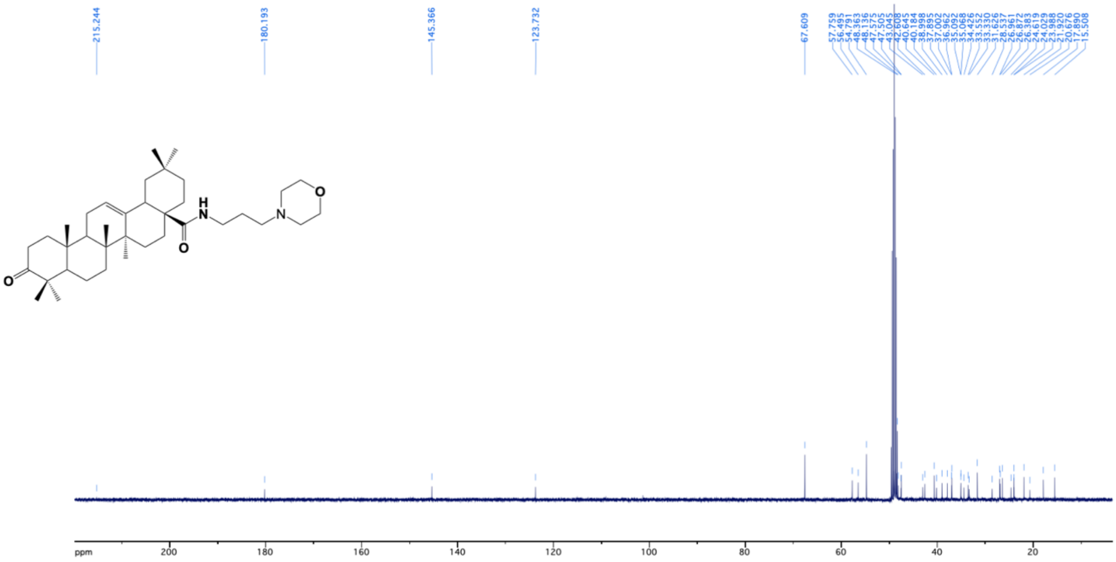

Figure S10. $^{13}$C NMR spectrum of compound **2e**

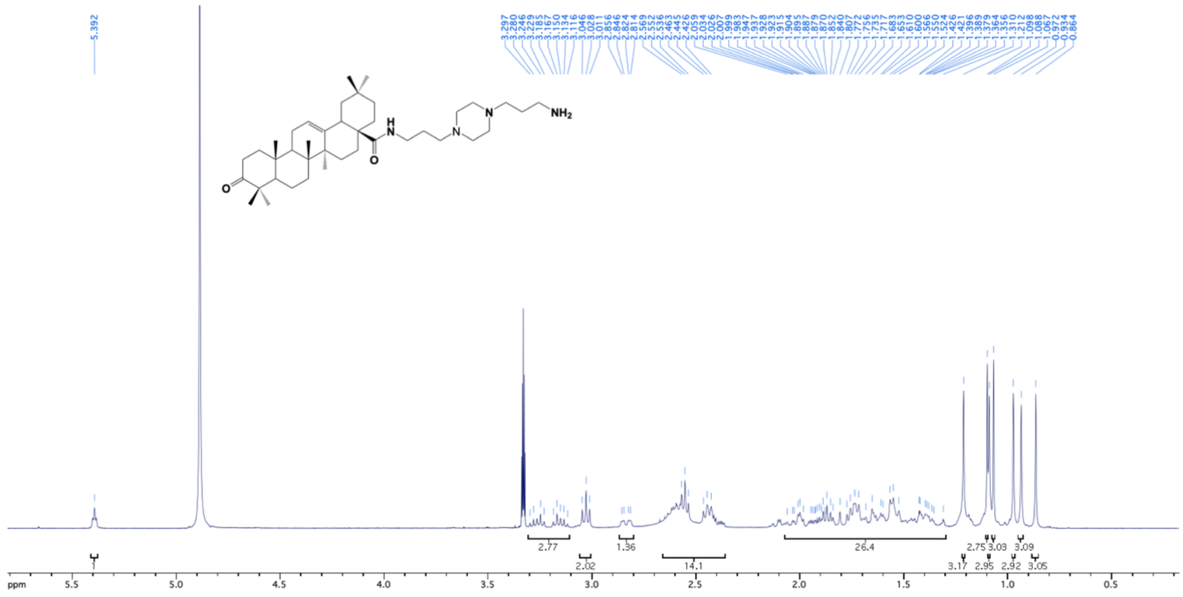

Figure S11. $^{1}$H NMR spectrum of compound **2f**

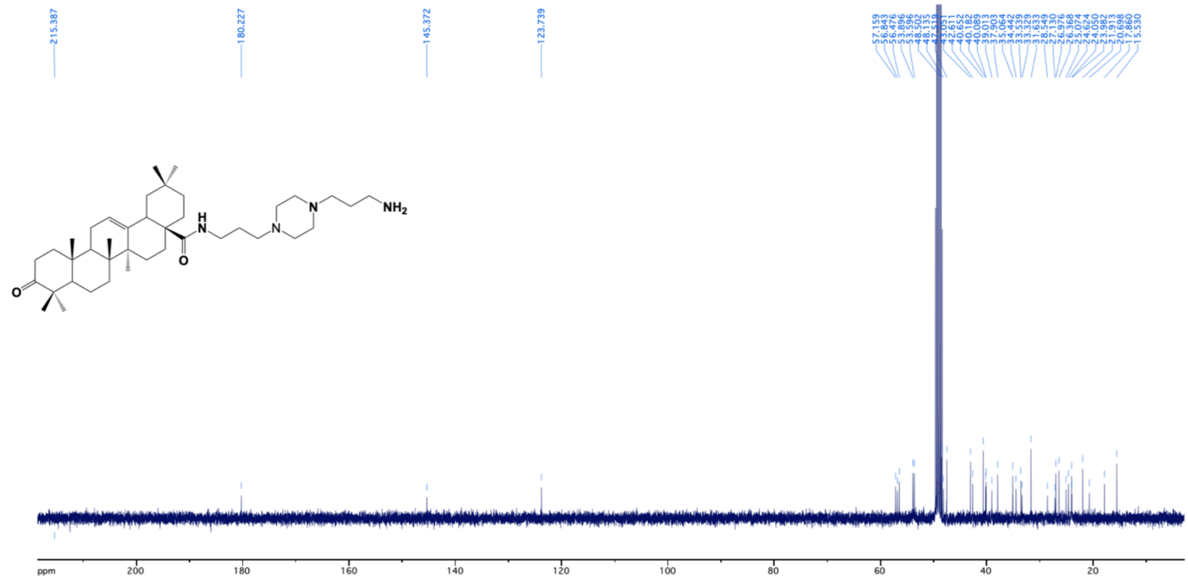

Figure S12. $^{13}$C NMR spectrum of compound **2f**

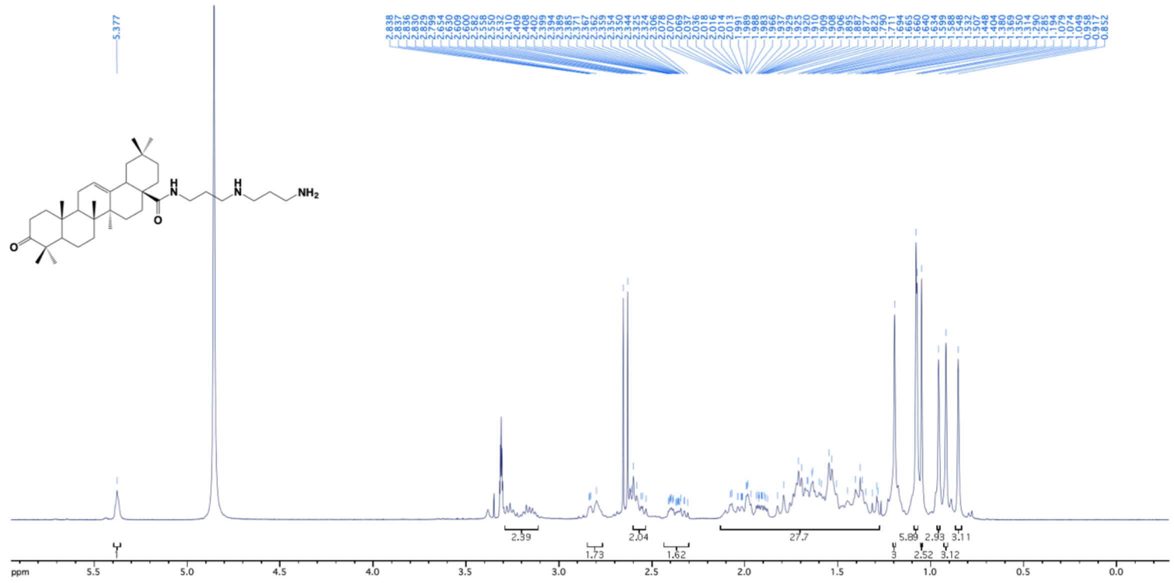

Figure S13. $^{1}H$ NMR spectrum of compound **2h**

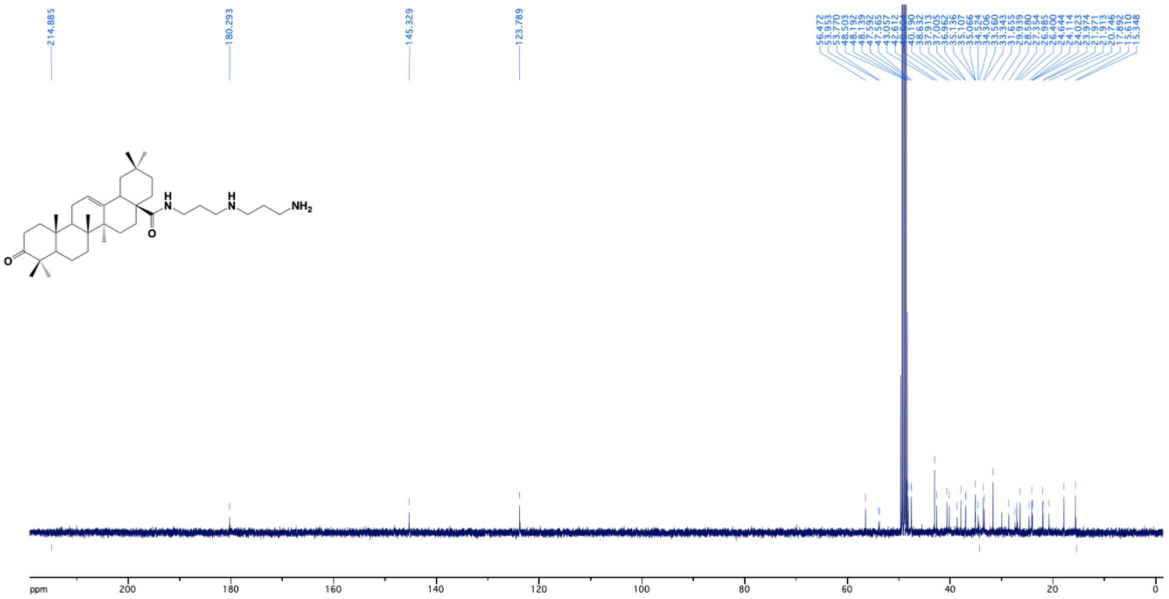

Figure S14. $^{13}C$ NMR spectrum of compound **2h**

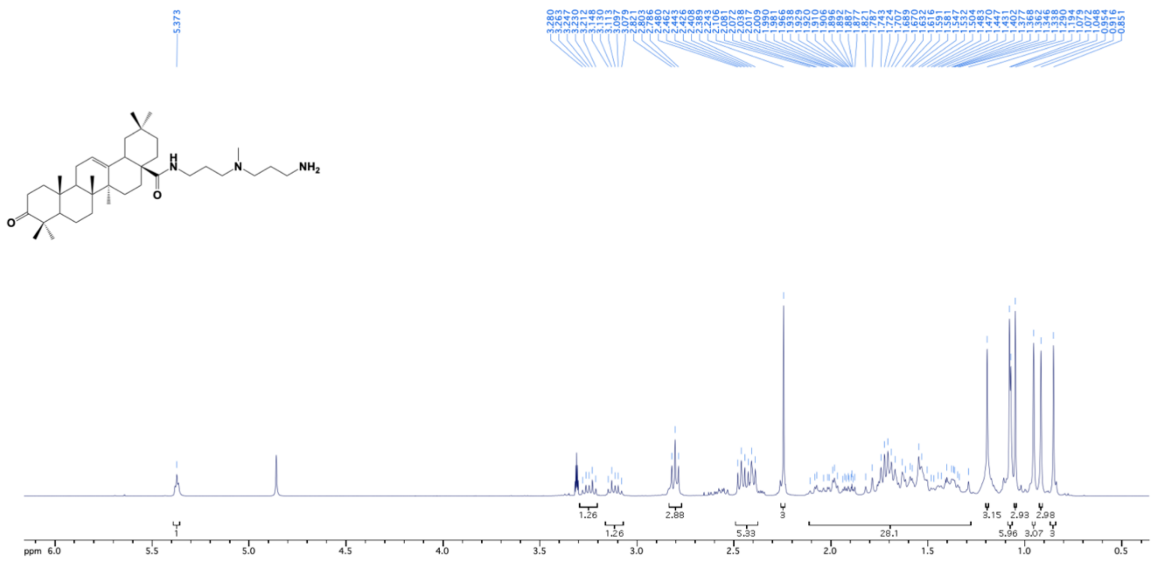


Figure S15. $^{1}$H NMR spectrum of compound **2i**

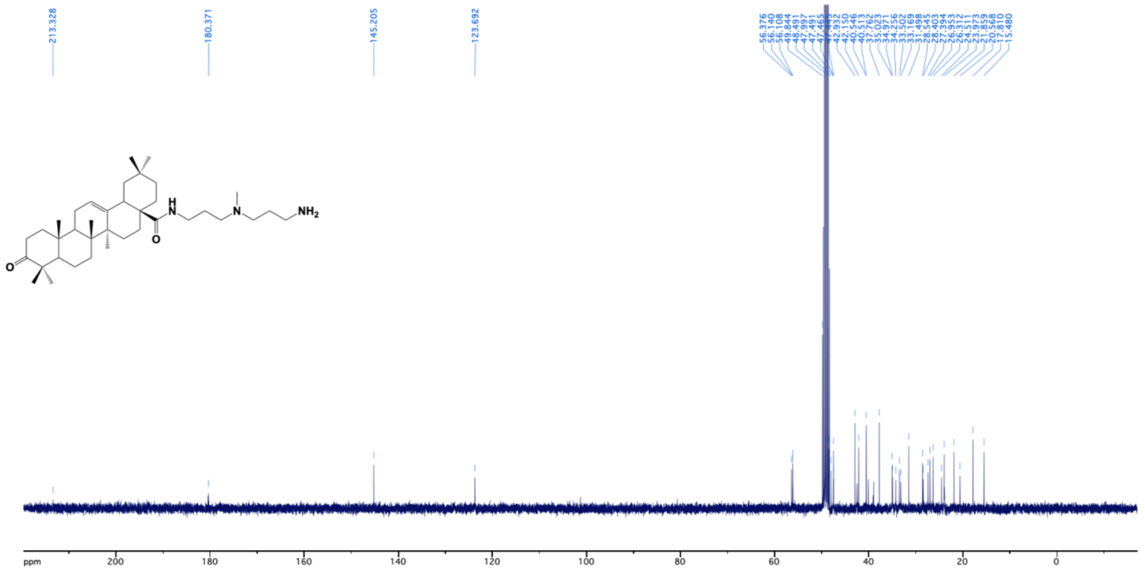


Figure S16. $^{13}$C NMR spectrum of compound **2i**

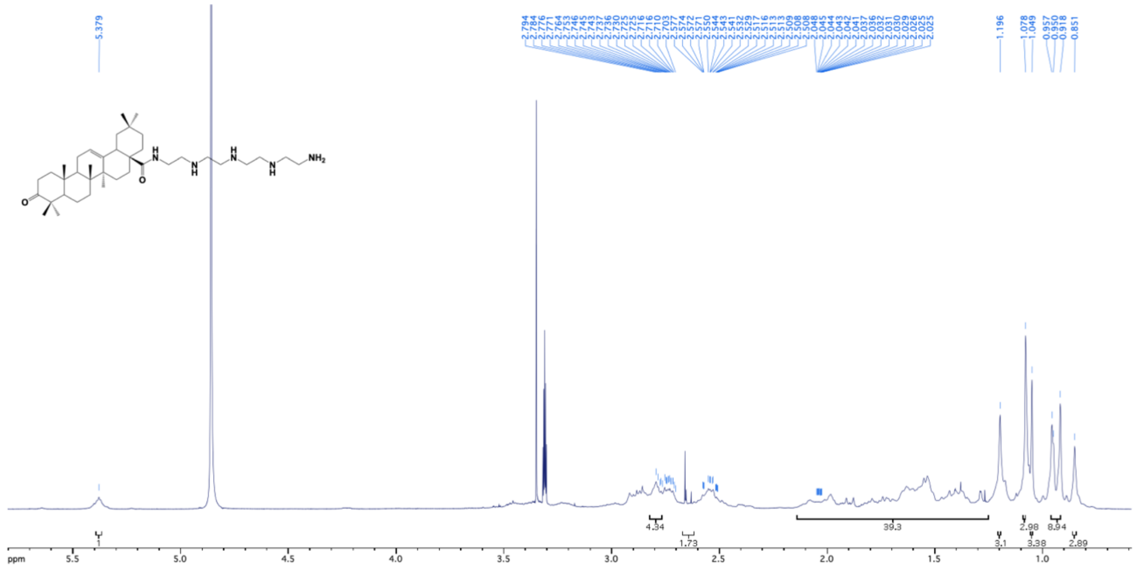

Figure S17. $^{1}$H NMR spectrum of compound **2j**

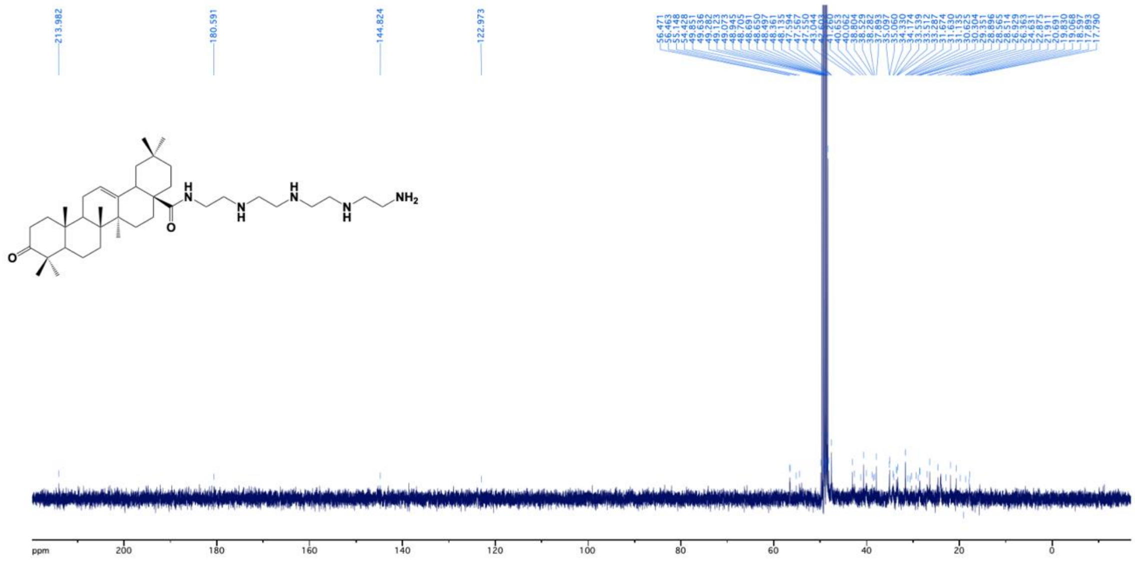

Figure S18. $^{13}$C NMR spectrum of compound **2j**

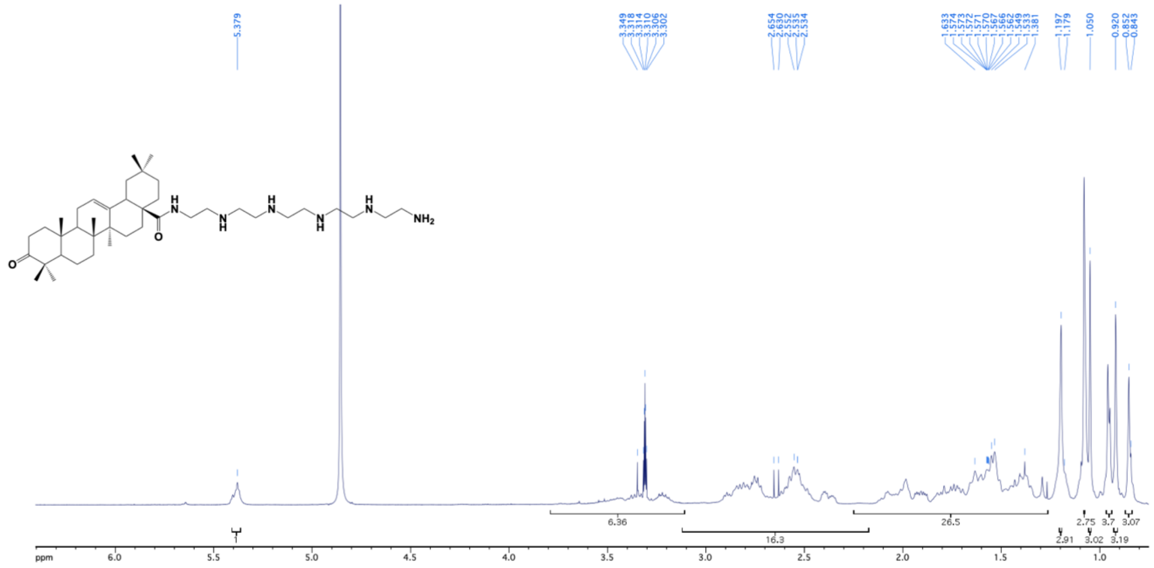

Figure S19. $^{1}H$ NMR spectrum of compound **2k**

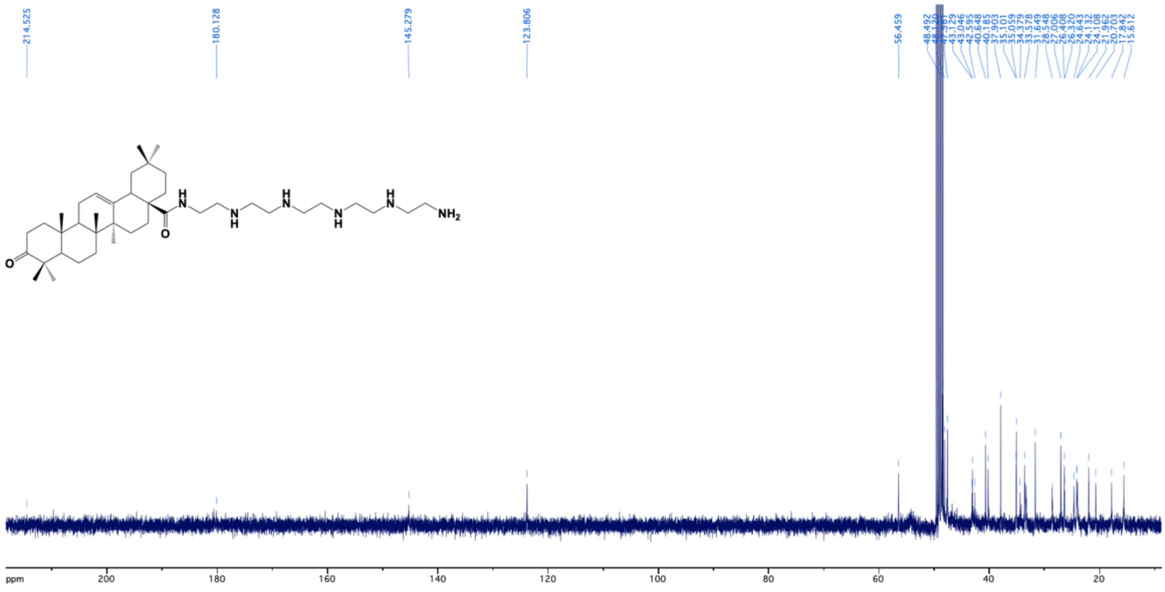

Figure S20. $^{13}C$ NMR spectrum of compound **2k**

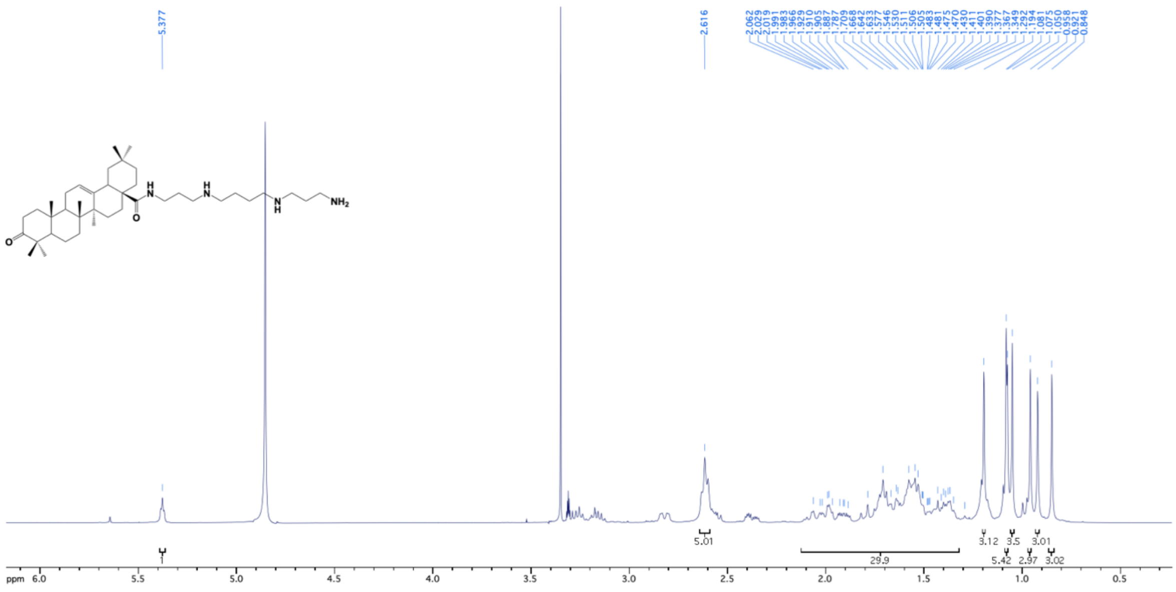

Figure S21. $^{1}$H NMR spectrum of compound **2l**

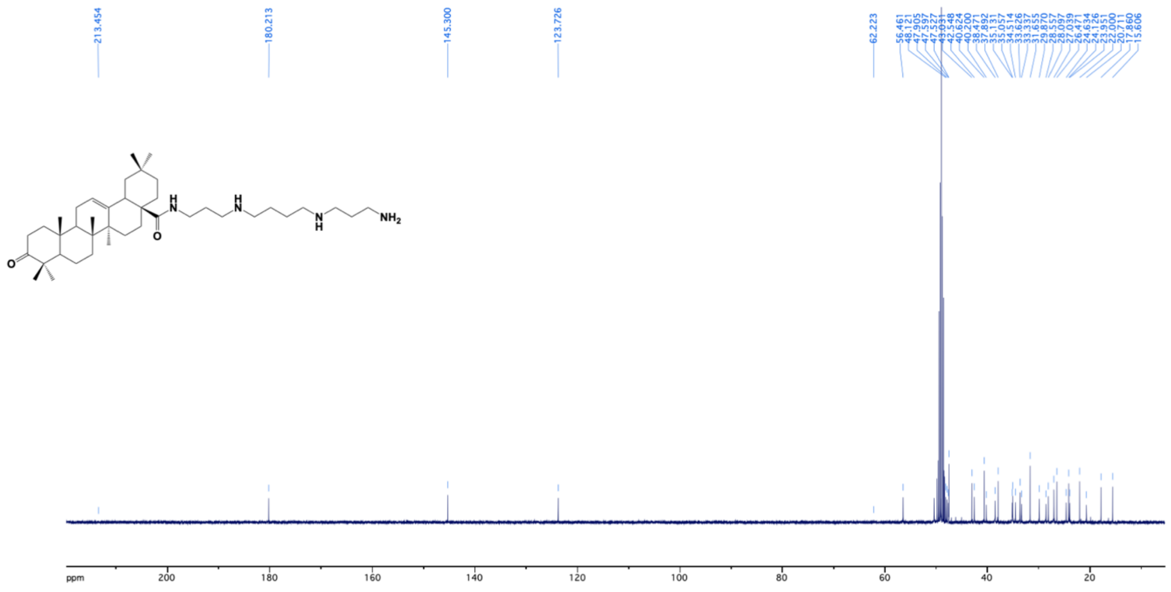

Figure S22. $^{13}$C NMR spectrum of compound **2l**

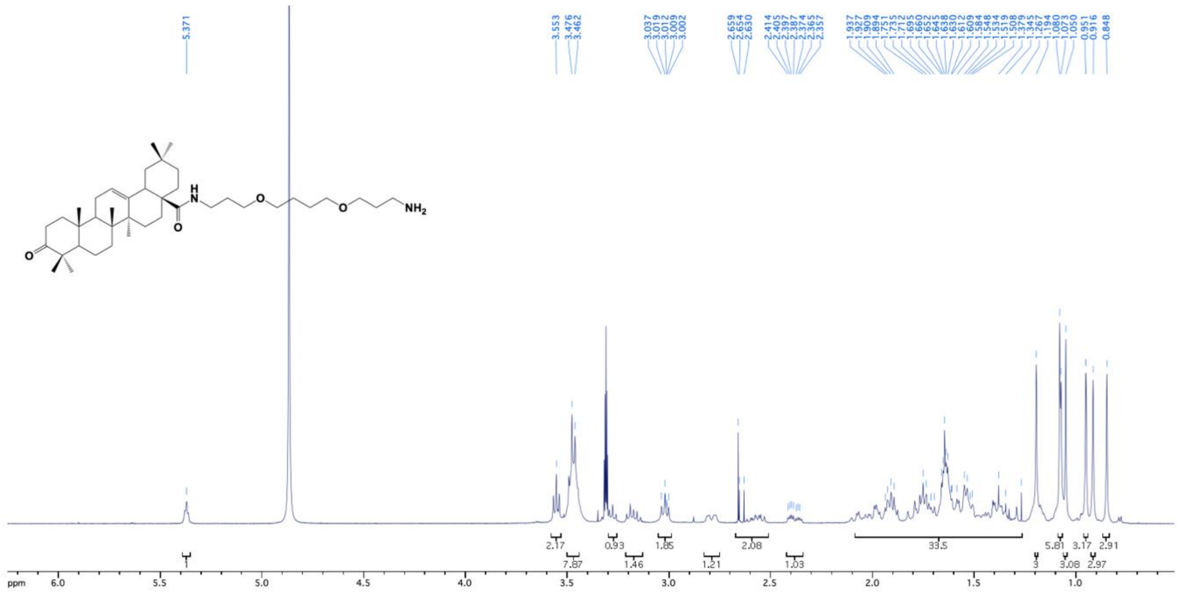

Figure S23. $^1$H NMR spectrum of compound **2m**

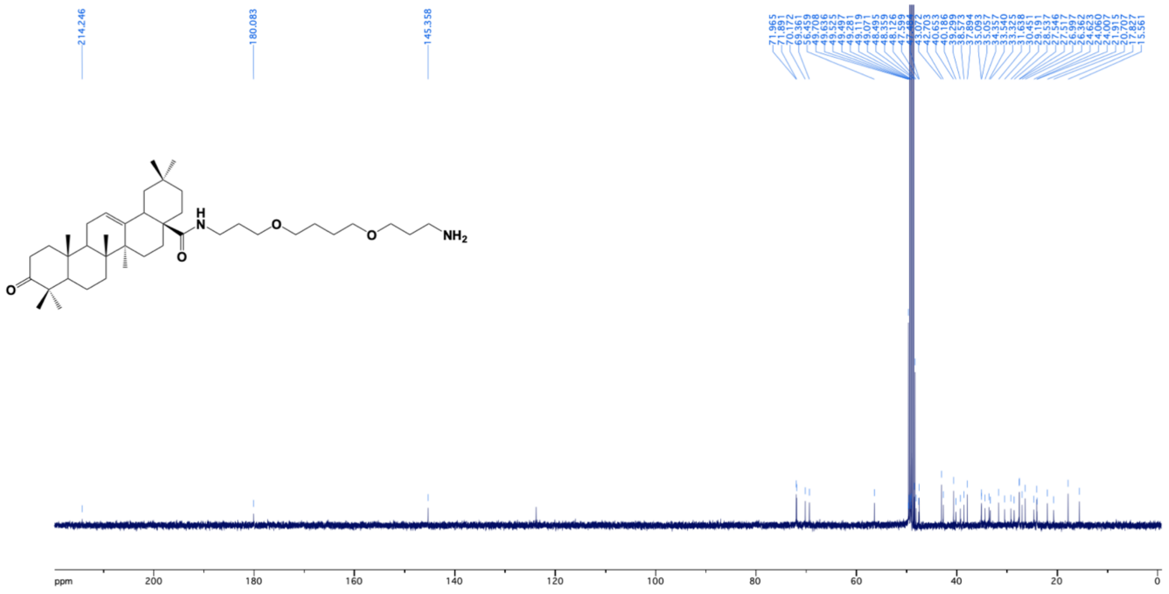

Figure S24. $^{13}$C NMR spectrum of compound **2m**

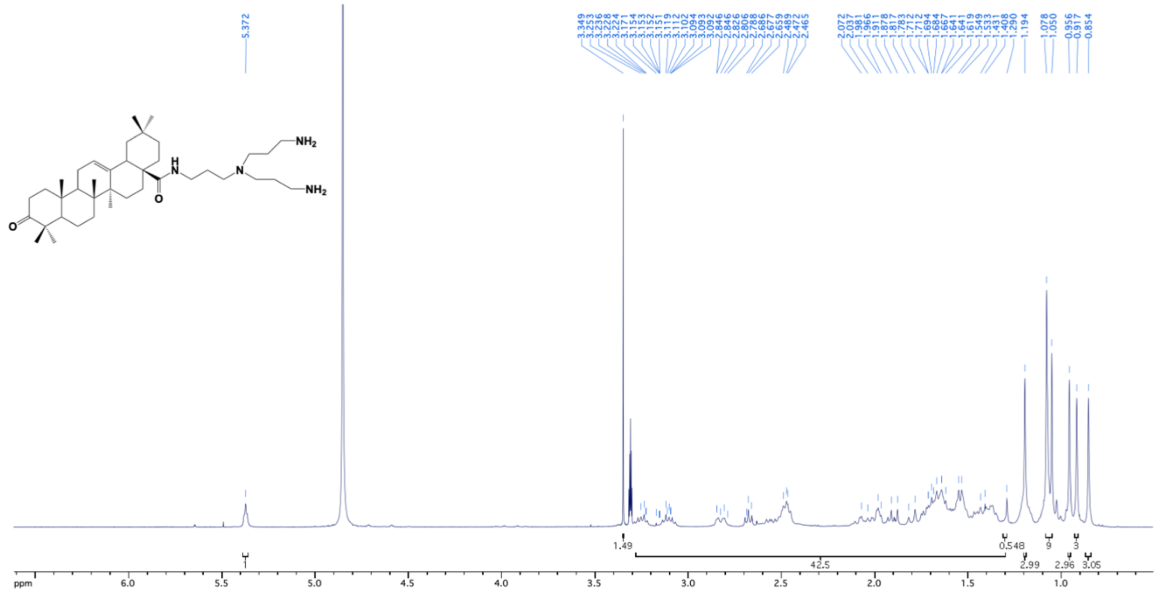


Figure S25. $^{1}$H NMR spectrum of compound **2n**

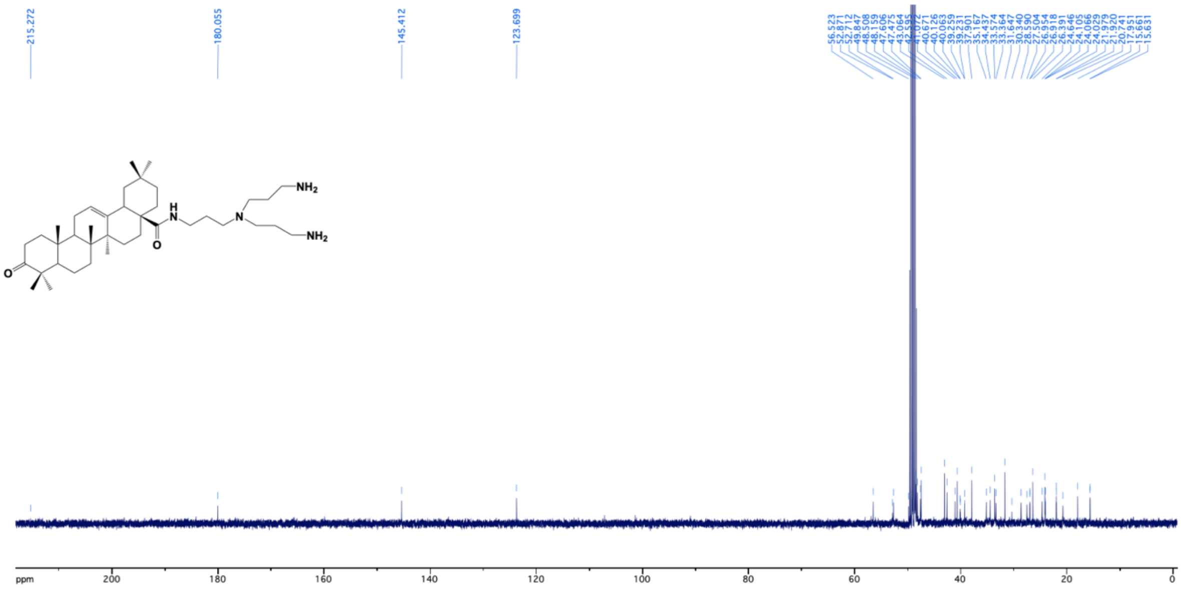


Figure S26. $^{13}$C NMR spectrum of compound **2n**

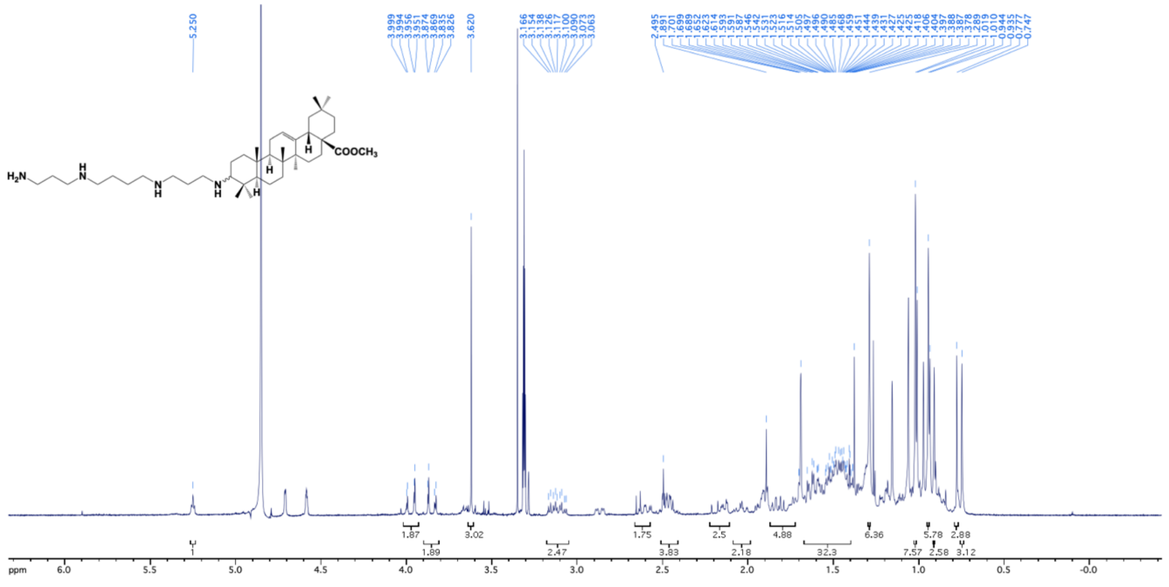

Figure S27. $^{1}$H NMR spectrum of compound **3**

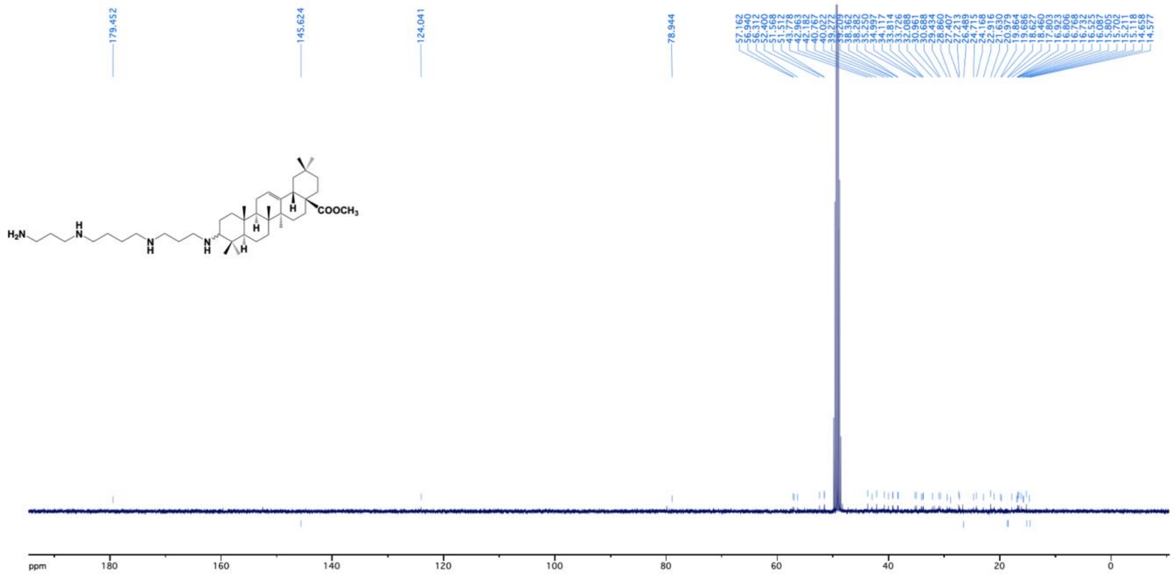

Figure S28. $^{13}$C NMR spectrum of compound **3**

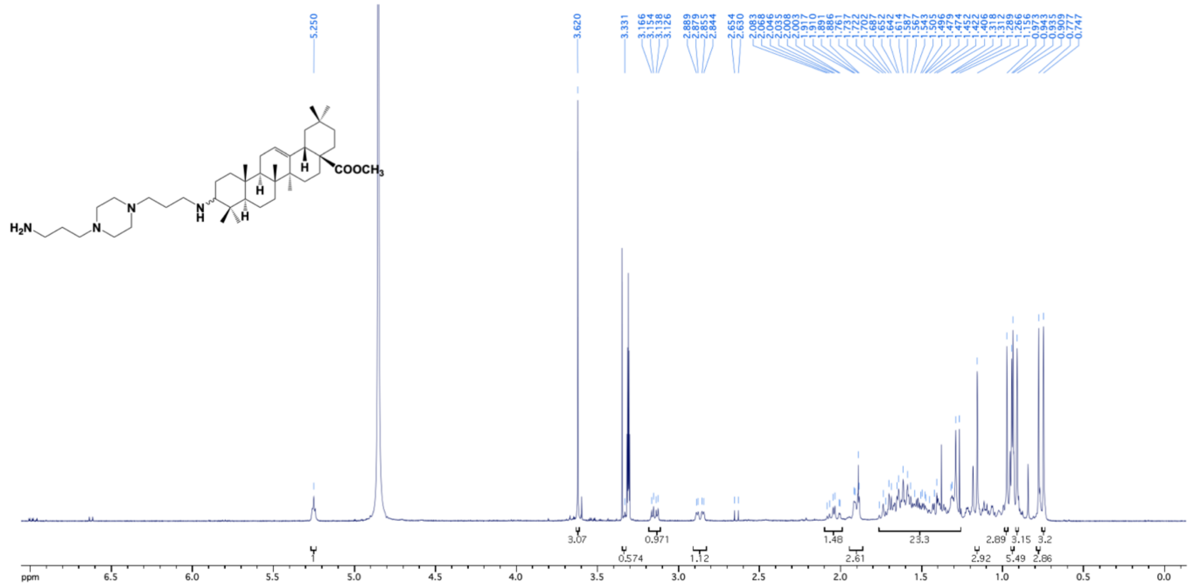

Figure S29. $^{1}$H NMR spectrum of compound **4**

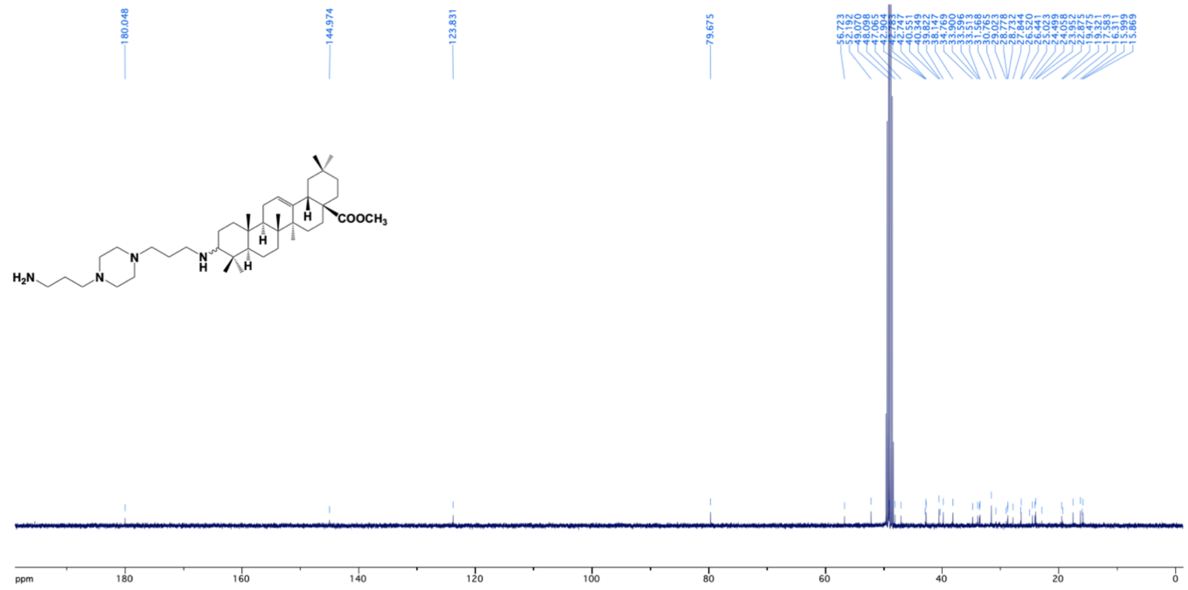

Figure S30. $^{13}$C NMR spectrum of compound **4**

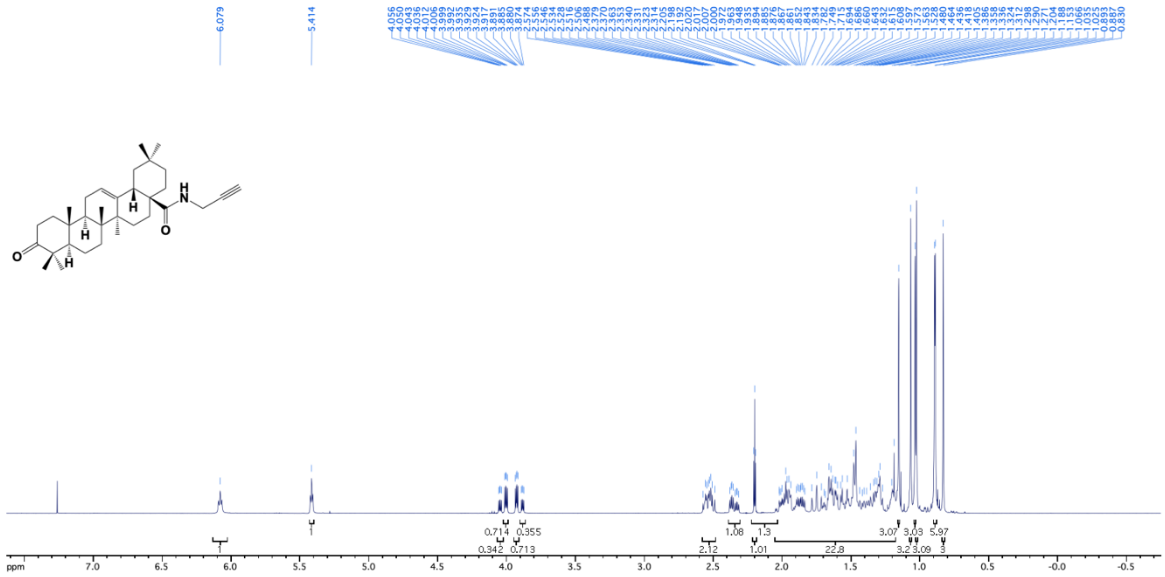


Figure S31. $^{1}$H NMR spectrum of compound **5**

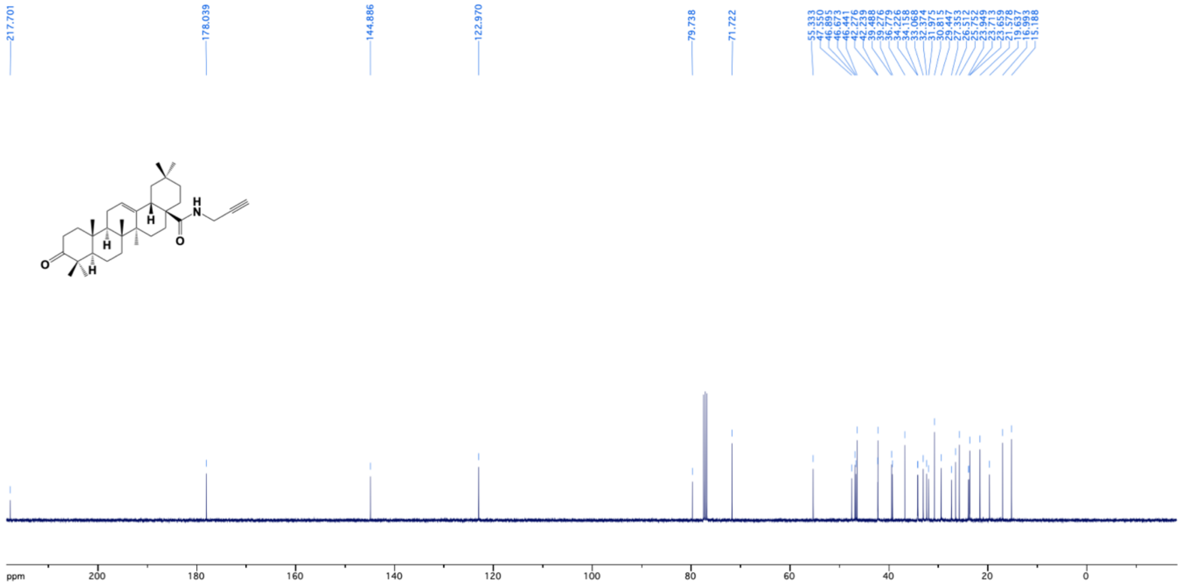


Figure S32. $^{13}$C NMR spectrum of compound **5**

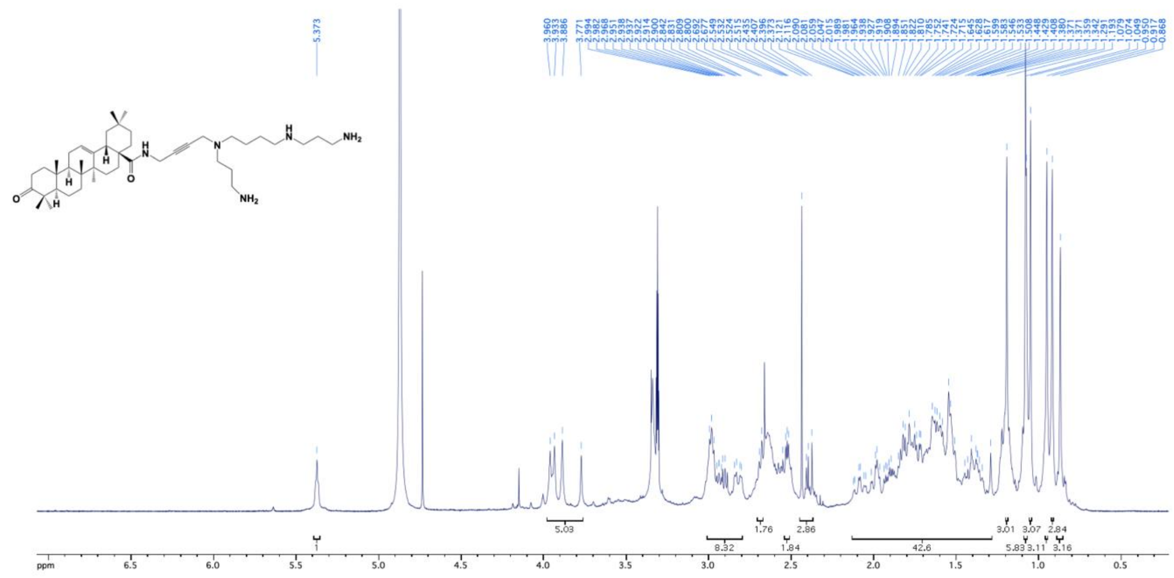

Figure S33. $^{1}$H NMR spectrum of compound **6**

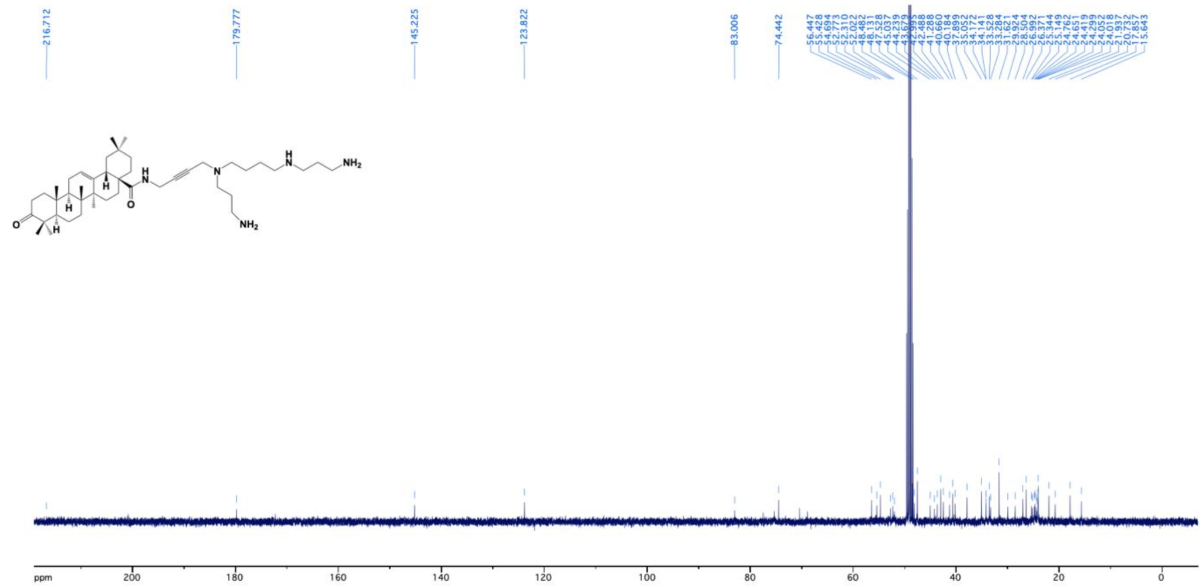

Figure S34. $^{13}$C NMR spectrum of compound **6**

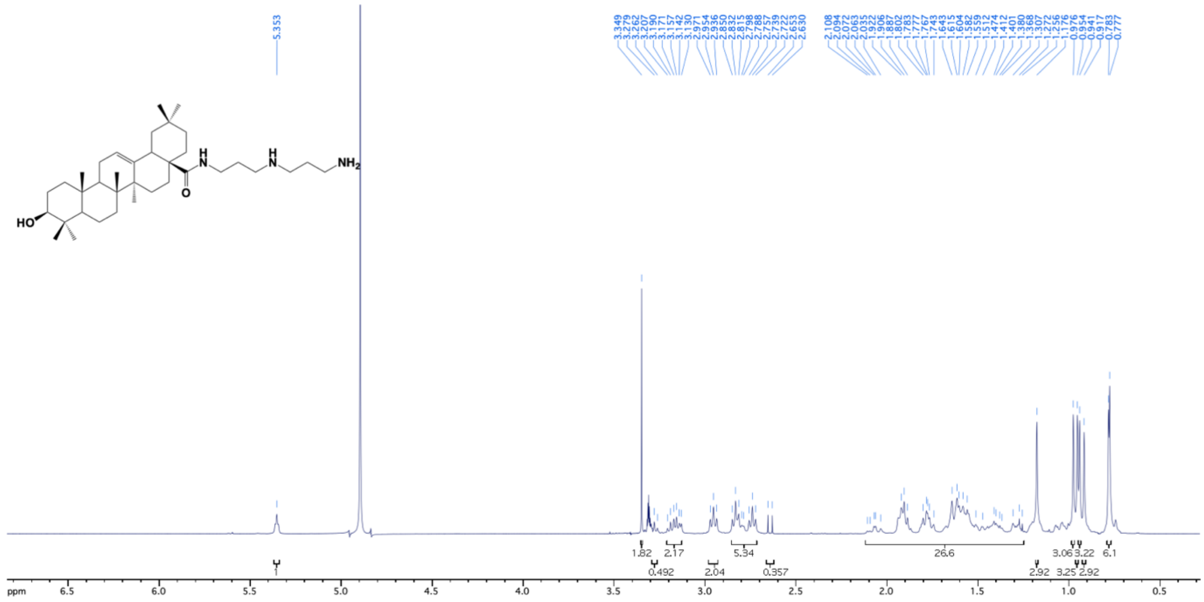


Figure S35. $^{1}$H NMR spectrum of compound **7a**

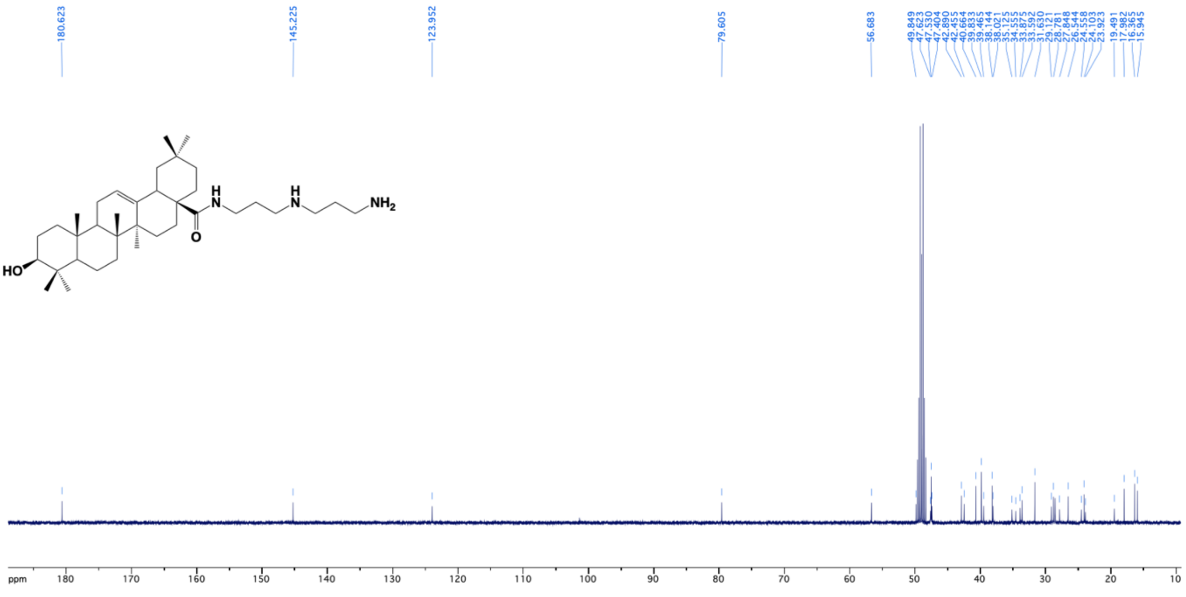


Figure S36. $^{13}$C NMR spectrum of compound **7a**

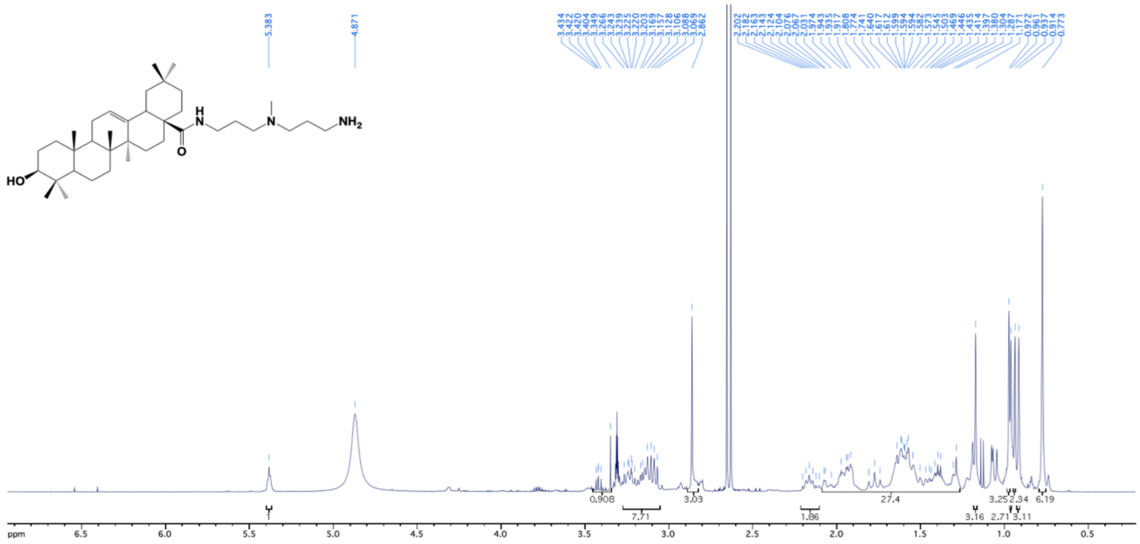

Figure S37. $^{1}$H NMR spectrum of compound **7b**

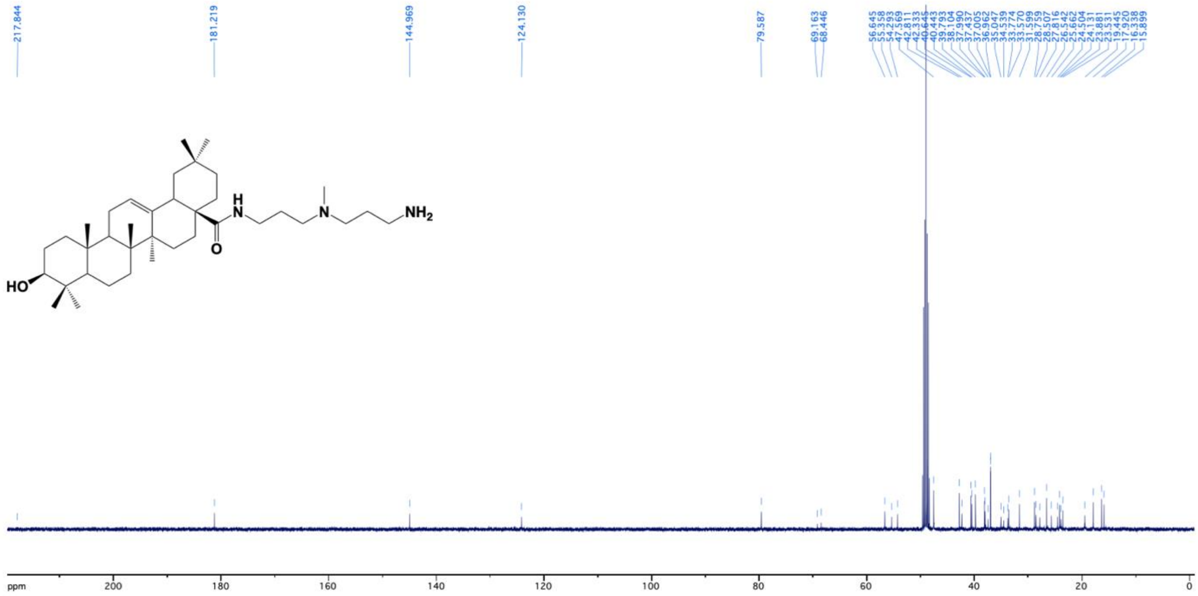

Figure S38. $^{13}$C NMR spectrum of compound **7b**

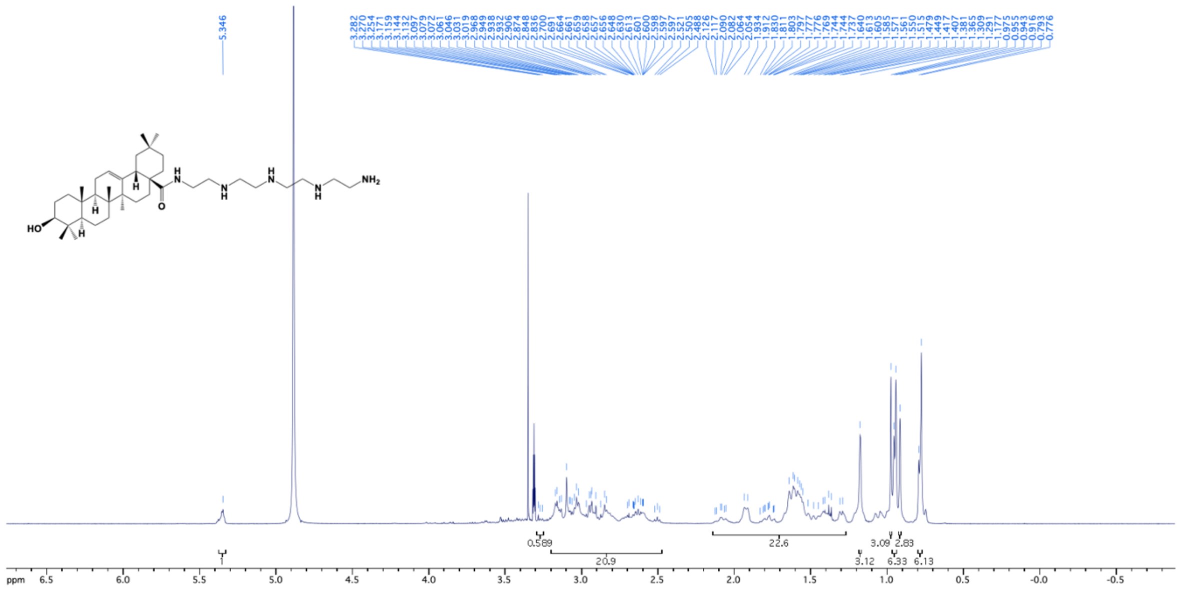


Figure S37. $^{1}$H NMR spectrum of compound **7c**

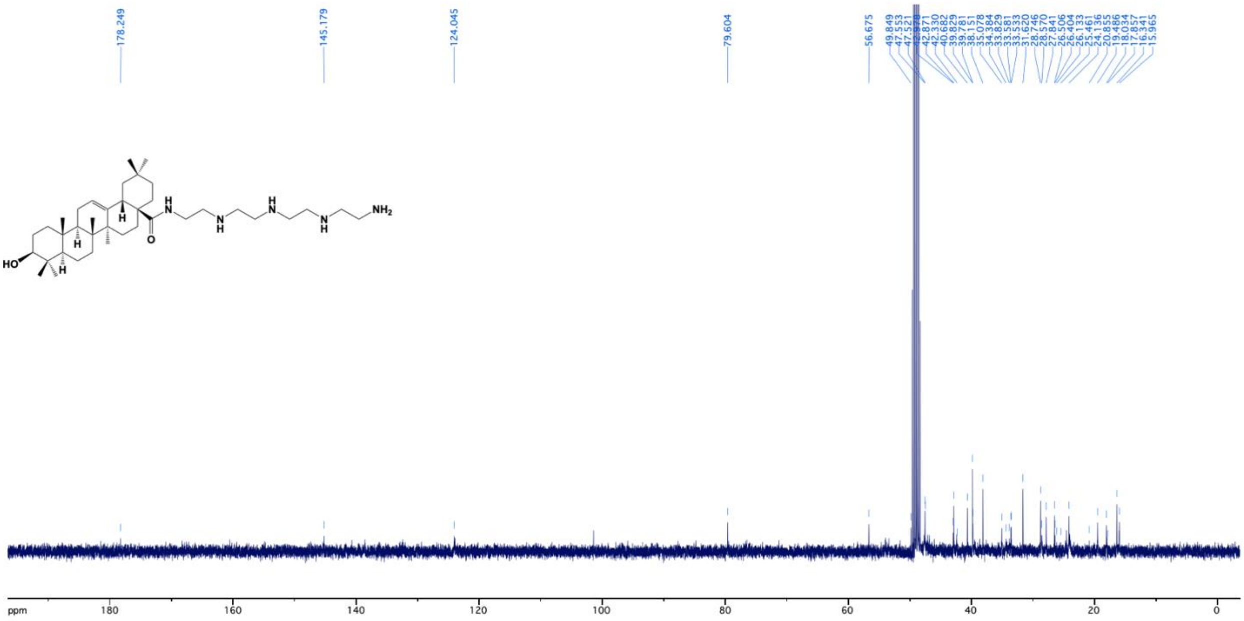


Figure S40. $^{13}$C NMR spectrum of compound **7c**
